# Executioner Caspase-3 and 7 Deficiency Reduces Myocyte Number in the Developing Mouse Heart

**DOI:** 10.1371/journal.pone.0131411

**Published:** 2015-06-29

**Authors:** Maria Cardona, Juan Antonio López, Anna Serafín, Anthony Rongvaux, Javier Inserte, David García-Dorado, Richard Flavell, Marta Llovera, Xavier Cañas, Jesús Vázquez, Daniel Sanchis

**Affiliations:** 1 Departament de Ciències Mèdiques Bàsiques, Universitat de Lleida–IRBLLEIDA, Av. Rovira Roure, 80, Lleida, 25198, Spain; 2 Centro Nacional de Investigaciones Cardiovasculares (CNIC), Melchor Fernández Almagro, 3, Madrid, 28029, Spain; 3 PCB-PRBB Animal Facility Alliance-Parc Científic de Barcelona, Baldiri Reixac, 4–6, Torre R, 4ª planta, Barcelona, 08028, Spain; 4 Department of Immunobiology and Howard Hughes Medical Institute, Yale University School of Medicine, 300 Cedar St., New Haven, CT 06520, United States of America; 5 Institut de Recerca Hospital Universitari Vall d’Hebron—UAB, Passeig de la Vall d’Hebron, 119, Barcelona, 08035, Spain; University of Cincinnati, College of Medicine, UNITED STATES

## Abstract

Executioner caspase-3 and -7 are proteases promoting cell death but non-apoptotic roles are being discovered. The heart expresses caspases only during development, suggesting they contribute to the organ maturation process. Therefore, we aimed at identifying novel functions of caspases in heart development. We induced simultaneous deletion of executioner caspase-3 and -7 in the mouse myocardium and studied its effects. Caspase knockout hearts are hypoplastic at birth, reaching normal weight progressively through myocyte hypertrophy. To identify the molecular pathways involved in these effects, we used microarray-based transcriptomics and multiplexed quantitative proteomics to compare wild type and executioner caspase-deficient myocardium at different developmental stages. Transcriptomics showed reduced expression of genes promoting DNA replication and cell cycle progression in the neonatal caspase-deficient heart suggesting reduced myocyte proliferation, and expression of non-cardiac isoforms of structural proteins in the adult null myocardium. Proteomics showed reduced abundance of proteins involved in oxidative phosphorylation accompanied by increased abundance of glycolytic enzymes underscoring retarded metabolic maturation of the caspase-null myocardium. Correlation between mRNA expression and protein abundance of relevant genes was confirmed, but transcriptomics and proteomics indentified complementary molecular pathways influenced by caspases in the developing heart. Forced expression of wild type or proteolytically inactive caspases in cultured cardiomyocytes induced expression of genes promoting cell division. The results reveal that executioner caspases can modulate heart’s cellularity and maturation during development, contributing novel information about caspase biology and heart development.

## Introduction

Caspases constitute a family of Cysteine-dependent Aspartate-directed proteases participating in apoptotic cell death [1]. By cleaving their targets, which include caspases themselves, caspases regulate many aspects from death signal propagation within the cell to genomic DNA degradation. Caspase gene mutation and deletion experiments unveiled their direct and evolutionally conserved implication in the control of embryonic cell death [2–4]. Interestingly, *in vivo* deletion of initiator caspase-8, which triggers the cell death intracellular signaling upon death receptor activation, or deletion of its regulators Cellular FADD-like IL-1β-converting enzyme-inhibitory protein (cFLIP) and FADD, lead to alterations in cardiac muscle differentiation [5–7]. Executioner caspase-3 and -7 function normally downstream of initiator caspases and their global deletion also impair cardiac muscle differentiation [8]. In addition, the effects of *in vitro* embryo culture in presence of caspase inhibitors suggested a role of caspase proteolytic activity in heart formation and muscle differentiation [9, 10].

Ubiquitous deletion of caspases prevented from identifying whether cardiac defects resulted from missing caspase activity in the developing myocardium or were secondary to defects in other tissues or cell types [11, 12]. Therefore, a conditional mouse knock out model is required to determine the role of caspases in the myocardium. Furthermore, the molecular and biochemical changes occurring in the heart underlying the morphological changes due to deletion of caspases or their regulators [5–7] has never been analyzed before.

To investigate the role of executioner caspases in cardiomyocytes *in vivo* we generated and studied cardiomyocyte-specific caspase-3 null / caspase-7 null mice in a C57BL/6J genotype. We demonstrate that caspase-3 and -7 influence cardiomyocyte number and proteome. Executioner caspase deficient hearts have broad changes in gene expression including decreased expression of genes involved in cell cycle progression. Quantitative proteomics suggested metabolic retardation occurring in the caspase-deficient heart and *in vitro* experiments confirmed the influence of caspases in the regulation of genes involved in cell division. Our results also show that caspase proteolytic activity is not required for regulating gene expression. Our findings complement previous knowledge on the functions of caspases in cardiac development and expand the understanding of executioner caspase activity.

## Materials and Methods

### Animal experimentation and ethics statement

The investigation with experimental animals was approved by the Experimental Animal Ethic Committee of the University of Lleida (codes CEEA06-01/10,07-01/10, 08-01/09 and 09-01/09) and conforms to the Guide for the Care and Use of Laboratory Animals, 8^th^ Edition, published in 2011 by the US National Institutes of Health. All mice (*Mus musculus*) had a C57BL/6J background and were housed at the Specific Pathogen Free facility of the Experimental Animal Platform—Parc Científic de Barcelona, lights on from 7 a.m. to 7 p.m., temperature = 18-22°C and 30–70% humidity. Animals are housed in Tecniplast GM500 cages (391x199x160 mm) never exceeding 5 adults / cage. Enriched environment includes autoclaved cellulose material. Animals are feeded 2914 diet (Irradiated Teklad Global 14% Protein Rodent Maintenance Diet, Harlan) and sterilized tap water, both ad libitum. Wellbeing of animals is monitored daily by visual inspection and each eight weeks pathogen analysis is monitored from sentinel animals following the standards determined by the Federation of European Laboratory Animal Science Association (FELASA). See ARRIVAL Guidelines Checklist (S1 File). Full caspase-7-deficient cardiac-specific caspase-3-deficient mouse strain was generated by crossing of caspase-7-deficient [8] and caspase-3 floxed mice [13] with the *Nkx2*.*5*::*Cre* transgenic mouse strain, a kind gift of Dr. Eric N. Olson (UT Southwestern Medical Center, Dallas, TX, USA) [14] (Figure A in S3 File). Genotypes were analyzed by PCR using tail DNA as a template, primer sequences can be found in S2 File and Figure B in S3 File. For each experiment requiring animals, they were randomly taken by the Facility staff from available individuals of the required age, gender and genotype. Animals were anesthetized with a lethal dose of inhaled isofluorane and decapitated within the Facility by expert staff. For protein and RNA extraction, hearts were dissected minced into small cubes, rinsed with cold phosphate buffered saline and snap-frozen into liquid nitrogen. For hypertrophy experiments, six 20-week-old mice per genotype were anesthetized with inhaled isofluorane (4% for induction and 2% for maintenance) for subcutaneous implantation of mini-osmotic pumps (Alzet, Durect Corporation) loaded with isoproterenol (Sigma-Aldrich), 8.8mg/kg/day, or saline vehicle, which were administered for one week [15]. Cardiac hypertrophy was evaluated by measuring heart weight *vs*. tibia length of sacrificed mice at the end of the treatment.

### Cell cultures

Rat neonatal cardiomyocytes were obtained from the ventricles of 1 to 4-day-old Sprague-Dawley pups housed in the conventional Animal Facility of the University of Lleida, as described previously [16]. Human Embryonic Kidney 293 cells (HEK293) were cultured and used both as positive control for cell death counting and for virus production as reported previously [17]. Staurosporine (Sigma-Aldrich) was added to the culture medium at 1µM and maintained for 16 hours before cell processing. Cell death was quantified using the Trypan Blue exclusion assay [18]. The number of independent experiments and intra-experiment replicas is specified in each figure legend.

### Transthoracic echocardiography

Echocardiographic measurements were performed using a Vivid portable ultrasound system with a ILS 12 MHz transducer (GE Healthcare) as described earlier [19]. Ejection fraction (EF), end-diastolic left ventricular internal diameter (LVEDD), end-systolic left ventricular internal diameter (LVESD), interventricular septum thickness (IVS) and posterior wall thickness (LVPW) were measured in M-mode recordings. Fractional shortening (FS) was calculated as ([LVEDD− LVESD]/LVEDD)×100. Nine adult wild type mice and 10 caspase-3 and -7 double knockout mice were used.

### Histological analysis of cell size in mouse hearts

Staining of heart sections with FITC-conjugated wheat germ agglutinin (WGA) was carried out as previously described [20]. In brief, animal’s codes were registered and mice were sacrificed by qualified technical personnel at the Animal Care Facility, hearts were fixed, included in paraffin blocks and 3 µm-thick slices were produced, stained with WGA-FITC and mounted. Slices obtained at two levels separated by 250–300 µm were stained for each heart to assure the presence of different myocytes. Each coverslip was identified with an Id code associated to the animal. Fluorescent microscopy images from 2 separate fields each covering 300 cells from each one of 6 animals per genotype, gender and age were obtained by a pathologist, who identified images with an Id code. The cross-sectional area of all cells appearing in full in the images was recorded using ImageJ software. Mean cross-sectional area was calculated for each heart and then the values were paired to the mouse of origin using the Id code and pooled per anatomical region (septum, ventricle), gender, age and genotype and the respective means were calculated.

### Quantification of cardiomyocyte cell number

Cardiomyocytes from wild type and caspase-3,7 double knockout hearts were isolated using the method described previously [21, 22]. Briefly, hearts were dissected and formol-fixed at 4°C followed by a treatment with 12.5M KOH overnight at 4°C. After a 10min vortex to dissociate the cells, cells were passed through a 250μm mesh, centrifuged, suspended in phosphate buffered saline (PBS) and cardiomyocytes were counted using a Neubauer chamber. Cardiomyocytes in the counting chamber were distinguished from fibroblasts and debris by cytoplasmic size and cell shape. The total number of cardiomyocytes from wild-type neonatal hearts was in the range of 10^6^, in accordance to reported values [22]. Six hearts per gender, age and genotype were processed and counted as explained in the figure legend.

### Preparation of subcellular extracts

Subcellular fractionation of rat neonatal cardiomyocytes was performed to analyze the localization of caspase-3 and caspase-7. Cytosolic fraction was performed as previously reported [16]. Mitochondrial fraction was obtained with the Mitochondria Isolation kit for cultured cells (Thermo Scientific) and nuclear fraction was obtained with the Nuclei EZ Prep Nuclei Isolation kit (Sigma-Aldrich), in both cases we followed the protocol determined by the supplier. Subcellular extract’s purity was checked by Western Blot using validated fraction markers as described in the figure legend.

### Lentiviral driven protein overexpression

Open reading frames (ORF) of human C*aspase-3* and *Caspase-7* (Source Bioscience) were amplified with primers adding EcoRI and XhoI sequences to 5’ and 3’ ends respectively, digested (Takara), purified (Nucleospin Extract II, Magerey-Nagel), subcloned into pcRII (Invitrogen) by using T4 DNAligase (Takara) and amplified in *E*. *coli* Stbl2 (Invitrogen). Then, a SfiI fragment containing the ORF was subcloned in the pEIGW-SK lentiviral vector (kind gift of Dr. Trono, Switzerland). Cysteine to Serine mutants from *Caspase-3* and *Caspase-7* were obtained using a Site-Directed Mutagenesis System (LifeTechnologies), and their ORF were sequence-verified. Lentiviruses were prepared in the HEK293T packing cell line as described previously [17]; cardiomyocytes were treated or processed after 4 days of transduction, as described elsewhere [17].

### RNA extraction, microarrays, reverse transcription and quantitative PCR

For heart and cardiomyocytes, total RNA was obtained from frozen tissues or cell pellets with the RNeasy Mini Kit (Qiagen). RNA concentration measurements and reverse transcription were done as described [17, 23]. For microarrays, RNA was extracted from either six wild-type (three males and three females) or double knockout hearts at P0-1 and at P30-35, totaling 24 samples. All heart RNA was from ventricle tissue only and passed several quality controls as scheduled by the facility. Detailed description of the procedures can be found in S2 File. Quantitative Real Time PCR was performed in a iCycler iQ PCR detection system and iQ v.3 and iQ v.5 software (BioRad), using the Taq Man Gene Expression Master Mix (Cat.N. 4369016) and the Gene Expression Assays from Applied Biosystems to amplify the transcripts of mouse *cdc6* (cell division cycle-6), *cenpa*, *fam107a*, *myh11* (Myosin heavy Chain 11), *nppa* (Natriuretic peptide precursor) and *slc2a4*; mouse and rat *ccne1* (Cyclin E1), *ndrg4*, *pold1* (DNA polymerase subunit delta) and *mef2a*; and rat *serpina3*, with simultaneous amplification of *gapdh* as loading control (Applied Biosystems). In each qPCR assay, a mean threshold cycle (Ct) was obtained from triplicates of two or three independent samples. The sample Ct was corrected using the Ct of the loading control (gapdh) to obtain the ΔCt value. Then, differences for each sample against a reference sample (e.g. neonatal values) were calculated as a final ΔCt = ΔCt_reference_- ΔCt_sample_. Relative transcript abundance was then calculated using final ΔCt as 2^ΔCt^. The number of independent samples and replicates is specified in the figure legends.

### Protein extraction, SDS-PAGE and Western Blot

Protein expression was analyzed in 40 μg of total protein extracts from cell cultures and tissues diluted in Tris-buffered 2% SDS solution at pH 6.8 and SDS-PAGE was performed as described [17]. Antibody specifications are described in S2 File. Western blots were performed as reported [17]. Densitometric quantification of the bands was performed with the ImageJ software from scanner images of film exposures in which bands were not saturated. Values were expressed as arbitrary units (AU) corresponding to the signal numerical value given by ImageJ. Densitometric values of the proteins of interest were not corrected by the loading control (GAPDH) given the high signal/expression of this enzyme compared with proteins regulating DNA biology and cell cycle. Procedures were conducted with extreme accuracy to assure equal inter- and intra-experiment loading for each sample, which was assessed by Coomassie staining of the gel. Values presented in the graphs correspond to means ± standard error media (SEM) of three independent set of samples blotted and quantified independently.

### Proteomics data collection and iTRAQ quantitative analysis

Ventricle samples from 1-day-old neonate, 1-month, 3-month and 8-month-old mice were analyzed. Two independent samples per age and genotype were pooled. Briefly, proteins were digested using the filter aided sample preparation (FASP) protocol and the resulting peptides labeled with iTRAQ, fractionated by cation exchange and analyzed by LC-MS/MS. Quantitative data were analyzed using statistical models developed in our laboratory. Detailed description of the procedures and references can be found in S2 File.

### Enzymatic caspase activity assay

Executioner caspase activity (Z-DEVD-AFC) was measured in neonatal cardiomyocytes, 293 cell extracts and heart samples as previously reported [16]. Protein concentration in the lysates was measured by the Bradford assay (BioRad) and equal loads of protein were mixed with the fluorogenic substrate (Calbiochem) at 50μM in 96-well plates. The plates were incubated at 37°C and fluorescence was read at intervals of 1h during the next 8h in the Infinite M200 fluorimeter (Tecan), with excitation filter set at 360nm and emission filter set at 530nm. Means ± SEM were calculated from three independent experiments performed in triplicates as specified in the figure legend.

### Statistical analysis

Student’s-t test was used for comparisons between wild type and knock out samples or scrambled/control and knockdown/overexpressing cell cultures, which involved experimental variables such as cell number, cell death, cell size, organ size, caspase enzymatic activity, mRNA expression using RT-qPCR and protein abundance analyzed by Western Blot. Statistical analysis details for the expression microarray and quantitative proteomics studies are specified in S2 File

## Results

### Generation and initial characterization of the cardiac-specific caspase-3 deficient / caspase-7 deficient mouse: caspases influence myocyte proliferation

**Fig 1:**
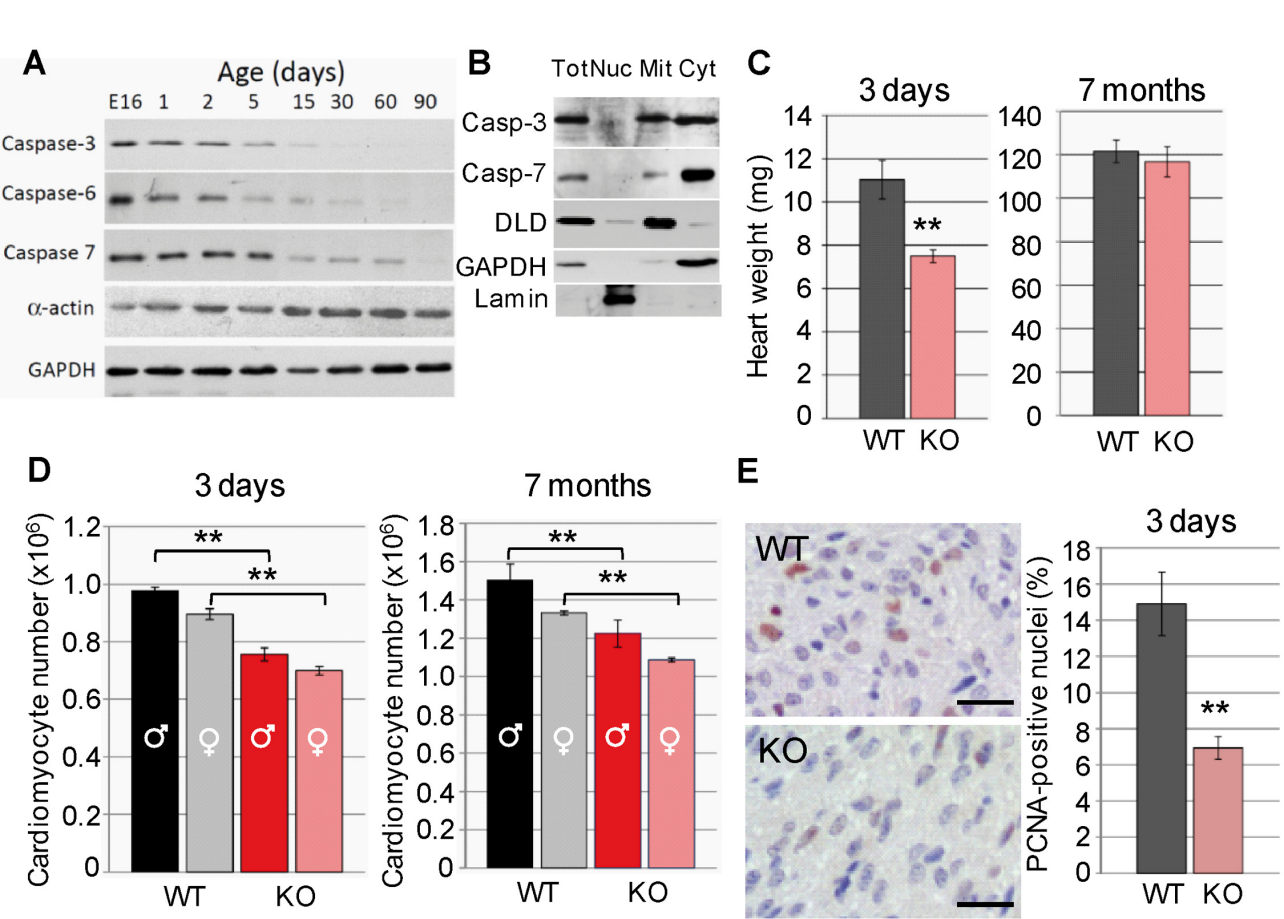
Executioner caspases are expressed in the developing heart and their conditional deletion in the myocardium reduces myocyte number at birth. **(A)** Expression of caspases-3,-6 and -7 in total protein extracts of hearts of mice at different ages from embryonic day 16 (E16) to postnatal day 30 (P30). Alpha-actin is a marker of myocyte differentiation. Glyceraldehyde-phosphate dehydrogenase (GAPDH) is a loading control. **(B)** Subcellular distribution of caspase-3 (Casp-3) and caspase-7 (Casp-7). Tot: total extract, Nuc: nucleus, Mit: mitochondria, Cyt: cytosol. DLD: dihydrolipoamide dehydrogenase (mitochondrial marker); GAPDH (cytosolic marker); lamin (nuclear marker). **(C)** Heart weight of 3-day-old and 7-month-old wild type (WT) and caspase-3 and caspase-7 knockout (KO) mice. N = 6 per age and genotype. **(D)** Cardiomyocyte number in hearts of male and female wild type (WT) and double mutant (KO) mice. N = 5 per gender, age and genotype. **(E)** PCNA (Proliferating Cell Nuclear Antigen) immunostaining of heart tissue slices from 3-day-old wild type and double mutant mice and graph showing percent PCNA^+^ nuclei in cardiomyocytes (fibroblasts have small and compressed cytosol). Bar: 20 µm. **, p<0.01 Student’s-t test KO vs. WT. Bars are means ± s.e.m.

Executioner caspase-3, -6 and -7 are expressed in the developing heart and are absent in the terminally differentiated myocardium (Fig 1A) suggesting that they are relevant during heart development. Progressive apoptotic gene silencing affects most of apoptotic genes and occurs soon after birth [17, 24], coinciding with cardiomyocyte exit from cell cycle [25]. Within cardiomyocytes, caspase-3 is expressed both in mitochondria and the cytosol, whereas caspase-7 is mostly cytosolic (Fig 1B) despite *in silico* search for mitochondria targeting sequences (MitoProt) [26] showed low probability of caspase-3 and -7 being exported to mitochondria (coefficient: 0.02 and 0.01, respectively, compared to 0.76 for the mitochondrial apoptotic nuclease EndoG).

Previous *in vivo* and *in vitro* studies suggest a role of executioner caspase-3 and -7 in heart development and myocyte differentiation [8, 10]. On the contrary, caspase-6 knockout mice have no overt cardiac alterations [27]. Although caspase-6 shares structural traits with caspase-3 and -7, it diverges from these caspases in terms of target peptide requirements and substrate affinity[1]. Whether caspase-3 and -7 are important in cardiomyocytes or abnormal cardiac development ensues from caspase deficiency in other cells *in vivo* is unknown. To determine the function of executioner caspases in the myocardium *in vivo* we designed a conditional knockout mouse in which caspase-3 gene deletion depends on *loxP* recombination driven by Cre recombinase expressed under the control of the *Nkx2*.*5* basal promoter-cardiac enhancer [14]. *Nkx2*.*5* promoter directs gene expression from the onset of cardiac commitment [14, 28]. *Caspase-7* was deleted ubiquitously because the lack of phenotype observed previously [8]. Caspase-3 and -7 double mutant mice were obtained by intercrossing *caspase-3* ^*lox/lox*^, *caspase-7* ^-/-^ and *Nkx2*.*5*::*Cre* mice (Figure A in S3 File). Genotyping reaction (S2 File) was designed to distinguish between *caspase-3* floxed (e.g. tail, cardiac fibroblasts) and knockout (myocardium) alleles. Western blot confirmed lack of caspase-7 expression in knockout mice and remnant caspase-3 expression in the neonatal heart (Figure B in S3 File).

Caspase-3 and -7 double mutant mice were born at Mendelian frequency (4.9% *vs*. expected 4.7%, n = 184) (Figure A in S3 File) and reached adulthood normally. However, the hearts of double knockout neonatal mice were 30% lighter than those of wild type mice, attaining normal weight by adulthood (Fig 1C). No differences were observed in heart anatomy between genotypes (data not shown). Cell counting from formol-fixed hearts [22] showed reduced cardiomyocyte number in double knockout mice at both ages, despite continued proliferation after birth in both genotypes (Fig 1D). The postnatal proliferation results (compare cell numbers in Fig 1D left and right graphs) agree with recent findings showing a cardiomyocyte proliferative burst in the postnatal heart [25], and also discard that normal heart weight of adult caspase knockout mice was due to increased late myocyte proliferation in the knockouts. Proliferating Cell Nuclear Antigen (PCNA) immunostaining of neonatal heart samples also agrees with lower number of cardiomyocytes involved in DNA replication in caspase-3 and-7 double knockout hearts (Fig 1E).

### Lack of executioner caspases reduces transcription of genes involved in DNA replication and cell cycle progression in the developing heart

**Fig 2:**
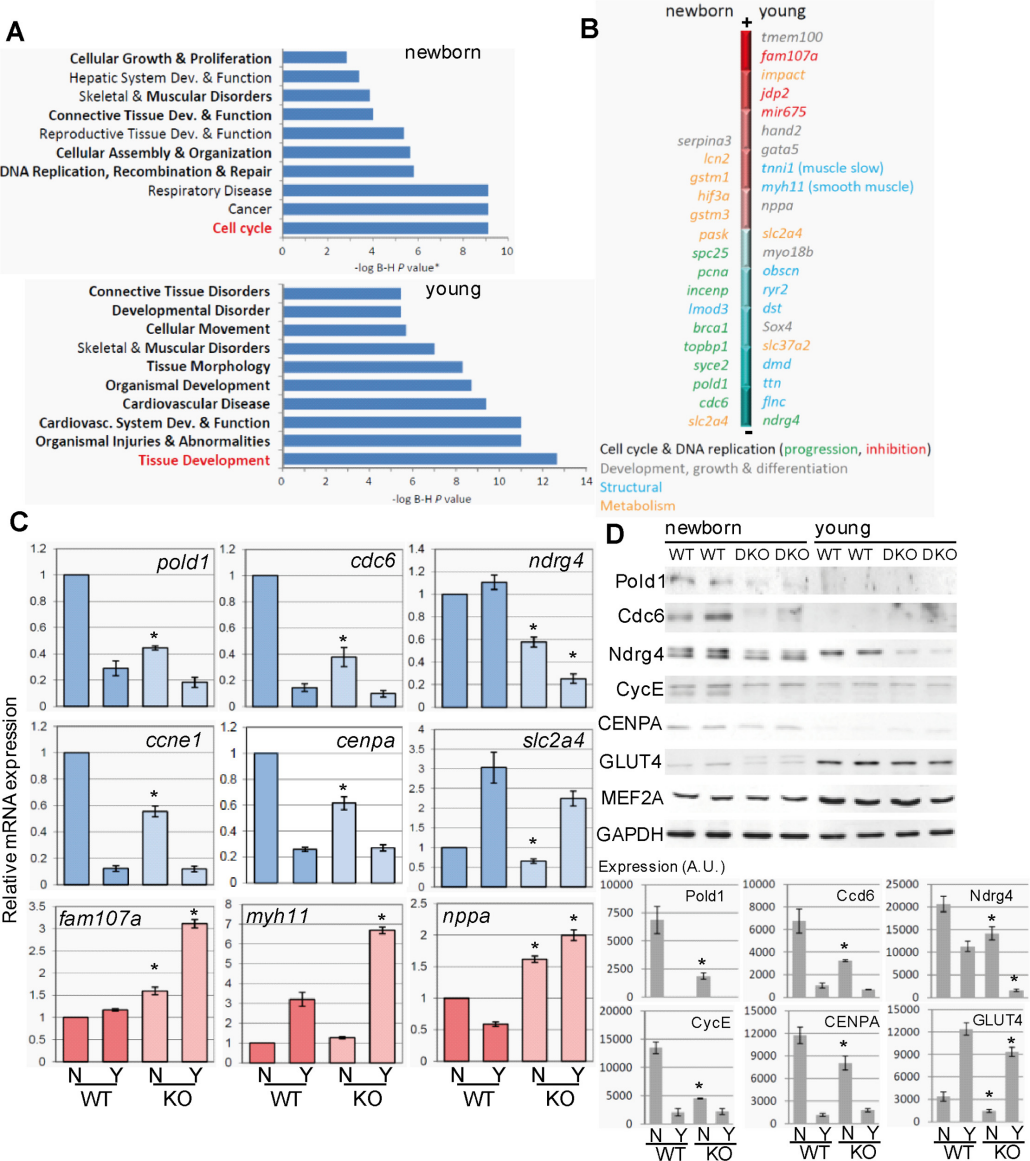
Executioner caspase deficiency in the developing myocardium triggers changes in gene expression in accordance to reduced myocyte proliferation. **(A)** Clustering of differentially expressed genes between wild type and caspase-3 and -7 double knockout mice by biological processes. Ploted is the–log of P values adjusted by the Benjamini and Hochberg method. Highlighted is the process most significantly affected in newborns and young mice. **(B)** Representation of top up-regulated genes (towards the red region of the bar) or down-regulated genes (towards the blue region of the bar) in newborn and young knockout mice, colored by molecular function. **(C)** Reverse-transcription/qPCR was performed to validate the microarray results for the expression of a set of genes downregulated (blue) or upregulated (red) in caspase KO hearts. N: newborn, Y: young. *, p<0.05, Student’s-t test KO *vs*. WT for the same age. n = 5–7 cardiac RNA extracts per age and genotype. **(D)** Changes of protein abundance for a set of the differentially expressed genes (selected by availability of antibodies with confirmed specificity). Upper panel: representative Western Blots (WB) featuring two WT and two KO independent cardiac protein extracts per age. Graphs: Densitometric analysis of WB. AU = Arbitrary units. *, p<0.05 KO *vs*. WT (Student’s-test) comparing the same age (N: newborn, Y: young). N = 6 per age and genotype. Mef2a expression is used as a control of unaffected gene and GAPDH is used as loading control. Bars are means ± s.e.m.

To determine the molecular changes underlying reduced cardiomyocyte number in the absence of executioner caspase expression, we performed a microarray-based gene expression analysis comparing wild type and caspase-3 and -7 double knockout hearts in newborns, an age in which wild type hearts still express caspases and in thirty-day-old mice, an age in which wild type hearts have downregulated caspase signaling and myocytes do not further divide). Gene functional categories most significantly affected in newborns by the lack of executioner caspases were those regulating DNA replication and cell cycle, whereas in young mice the most affected genes were those involved in tissue development (Fig 2A). Detailed inspection of transcription changes showed that newborn caspase-deficient myocardium transcribed abnormally low levels of genes coding for key proteins involved in DNA replication, recombination and repair (e.g. *pold1* coding for DNA polymerase subunit delta, *topbp1* coding for DNA topoisomerase-2 binding protein-1; *BRCA1*, *PCNA*, etc.), centromere and kinetochore formation (e.g. *CENP* genes, *INCEMP*, *auroraB*, etc.) and cell cycle regulation (e.g. *cell division cycle-6*, *ccne1* coding for cyclin-E), as well as some genes involved in metabolism (e.g. *slc2a4* coding for GLUT4, and *hif3a*, among others) (Fig 2B). In young hearts, lack of executioner caspases during development resulted in abnormally high expression of genes involved in cell cycle inhibition (such as *fam107a*, which was also increased in newborn knockouts), genes coding for non-cardiac isoforms of sarcomeric proteins (*myosin heavy chain-11* and *tropomyosin-2*), as well as genes involved in embryonic development (*hand2*, *gata5*) and also resulted in slightly reduced expression of genes coding for components of the contraction machinery (e.g. *titin*, *dystrophin*, *obscurin*) (Fig 2B, Figure C in S3 File). Gene expression changes detected by the microarray study were confirmed using specific quantitative PCR (qPCR) assays for a set of key genes involved in the above mentioned functions (Fig 2C). Of note, the mRNA of natriuretic peptide A (*nppa*), which is a marker of myocyte hypertrophy [29], was highly expressed in caspase-3 and -7 double mutant hearts and the opposite effect was observed for ndrg4, which is involved in cardiomyocyte proliferation [30]. Western blotting analysis revealed that changes in expression of selected proteins were coherent with the alterations observed in mRNA abundance related to age and genotype (Fig 2D). The expression changes observed in double mutant hearts were present yet modest in caspase-3 deficient hearts, while lack of caspase-7 did not affect the expression of any gene checked (Figure D in S3 File). Gene expression was also unaffected by Cre expression (Figure D in S3 File), discarding that changes observed were due to expression of the recombinase used to interrupt *caspase-3*. Taken together, the results obtained by the microarray-based gene expression study show that lack of caspase-3 and -7 in the developing myocardium deeply modifies the expression of genes involved in DNA replication, recombination and repair, chromatin organization and cell cycle progression in accordance with reduced cell proliferation. The results also show increased expression of non-muscular and smooth muscle structural gene isoforms in hearts of young caspase knockouts, in accordance with the gene expression changes occurring during hypertrophy previously described elsewhere [31].

### Proteomic profiling suggests changes in energy metabolism in the caspase-deficient heart

**Fig 3:**
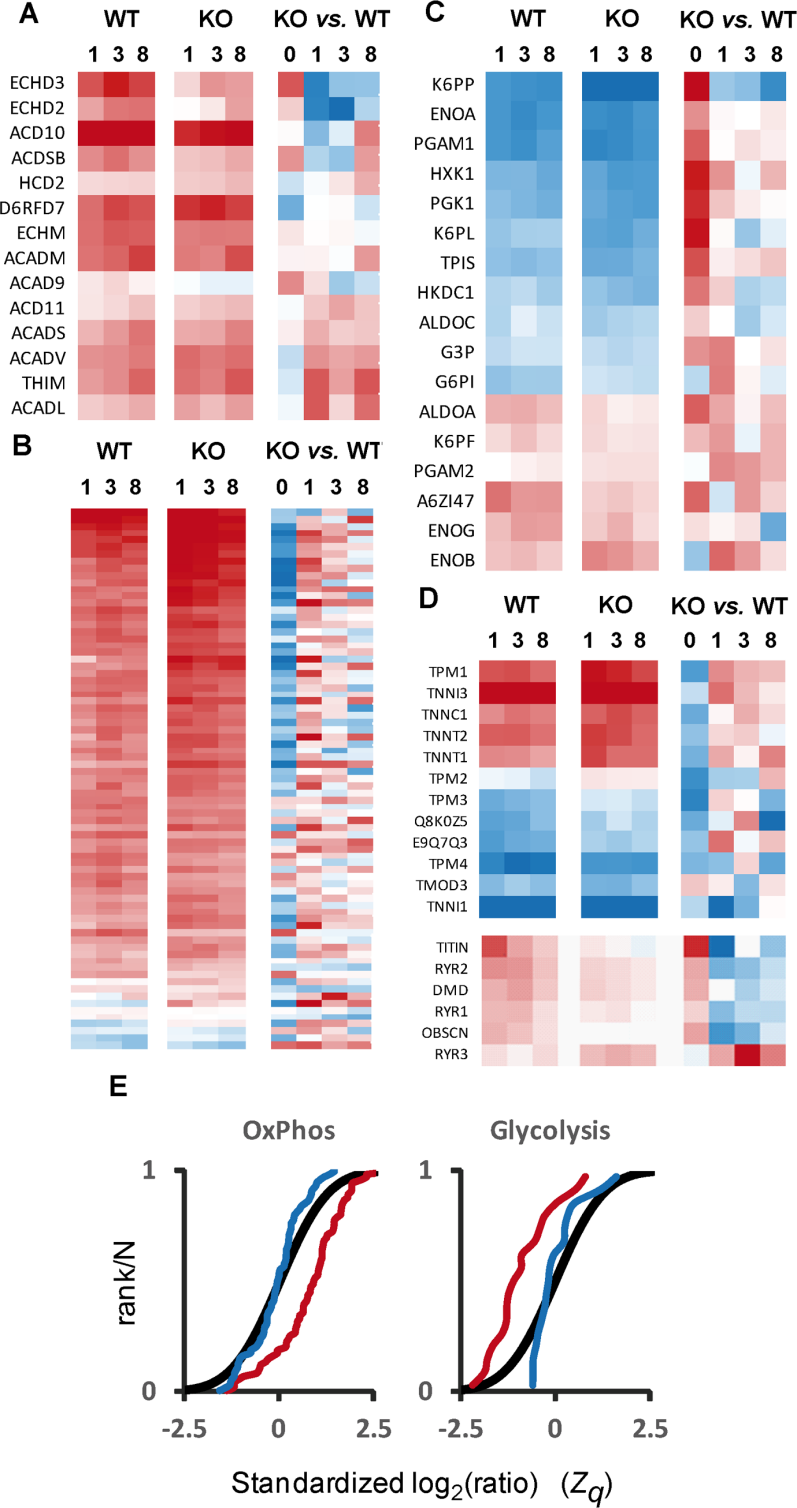
Quantitative proteomics analysis indicates metabolic changes due to executioner caspase deficiency in accordance with reduced myocyte maturation. **(A-D)** Relative abundance profiles of selected proteins along development (0, 1, 3 and 8-month-old) in both wild type (WT) and caspase-3 and -7 knockout (KO) animals; in the left and middle columns, the changes are expressed separately for KO and WT animals in relation to the abundances at t = 0, while in the rightmost column the abundance of proteins in the KO are compared with that of the WT animals at each time point. Selected proteins include those belonging to beta-oxidation **(A)**, oxidative phosphorylation complexes, whose identification can be found in Figure G in S3 File. **(B)**, glycolysis **(C)** and structural and contractile proteins **(D)**; in the case of glycolysis, for simplicity only the main or housekeeping isoforms are depicted. Red squares indicate increased abundance vs. neonatal values in columns referred to age (left and middle), and increased abundance vs. WT in columns comparing genotypes (right), while blue squares indicate decrease in relative protein abundance. The distribution of standardized log2-ratios (KO/WT) of proteins belonging to oxidative phosphorylation complexes and glycolysis in neonates (red lines) and in adults (blue lines) and the null hypothesis distribution (black lines) are compared in **(E)**, where a trend to the left denotes increased abundance and a trend to the right indicates lower abundance vs. the null hypothesis.

To gain further insight on the changes of protein abundance induced by executioner caspase deficiency along developmental stages, we conducted a high-throughput quantitative proteomics study. We used isobaric 8-plex peptide labeling (iTRAQ) followed by peptide prefractionation before mass spectrometry analysis to make a simultaneous quantitation of the deep cardiac proteome of wild type and executioner caspase knockout mice at four ages (1-day-old and 1, 3 and 8-month-old animals). The quantitative data were then analyzed using the WSPP model (S2 File), and were found to follow with good accuracy the predictions of the null hypothesis at the spectrum, peptide and protein levels (Figure E in S3 File). Comparative analysis of the dynamic behavior of proteins along time (Fig 3A–3D) revealed a switch from glycolytic to fatty acid oxidative metabolism through postnatal heart maturation [32] in both genotypes. This was evident by the increase in abundance of enzymes from the β-oxidation pathway (Fig 3A left and middle columns) and of components of oxidative phosphorylation complexes (Fig 3B left and middle columns).Two populations of glycolytic enzymes were defined in terms of their relative abundance along time (Fig 3C left and middle columns). On the one hand, eleven proteins were downregulated along time (blue) compared to postnatal expression (time 0, not shown). These proteins are coded by major glycolytic genes and their abundance decreased with age. On the other hand, the abundance of six proteins increased with age (red). From the later population, five proteins (ALDOA, a variant of ALDOA identified as A6ZI47,K6PF,PGAM and ENOB) correspond to muscle isoforms of glycolytic genes enriched in the adult muscle, despite reduced glycolytic activity compared to the embryo [32]. Despite this trend was observed in both genotypes, the majority of oxidative phosphorylation proteins were less abundant and glycolytic proteins were more abundant in the caspase-deficient myocardium at birth and this difference attenuated along time (Fig 3B and 3C right columns). We also observed that the abundance of the sarcomeric proteins troponin, tropomyosin and tropomodulin was lower in the knockout hearts than in the wild type hearts at birth, tending to reach similar levels in the adult (Fig 3D upper columns). On the contrary, other sarcomeric proteins including dystrophin, titin, obscurin and the muscle-specific ryanodine receptors 1 and 2 were relatively more abundant in the knockout hearts at birth but did not follow the normal developmental upregulation, resulting in abnormally low expression in these hearts at adulthood (Fig 3D lower rows). Of note the non-muscle isoform ryanodine receptor-3 (RYR3) was upregulated in the knockout hearts. To further illustrate the differences due to genotype, the distribution of standardized log2-ratios (WT/KO) of proteins from these categories was compared at 1 day and 8 months (Fig 3E). The sigmoid graphs show how absence of executioner caspases in postnatal hearts, when wild type hearts still express caspases, produced a protein distribution consistent with a more glycolytic and less oxidative metabolism than wild type hearts, suggesting metabolic retardation. However, no differences become appreciable at adulthood, suggesting adaptation towards an adult genotype, in which caspases are normally absent in the myocardium (Fig 1A and references [17, 24]). Overall, these results reveal that the caspase-deficient myocardium suffers changes in metabolic enzymes and proteins involved in contraction suggesting a relative retardation and alteration of cardiomyocyte maturation.

### Cardiomyocyte hypertrophy occurs in the heart deficient for caspase-3 and -7

**Fig 4:**
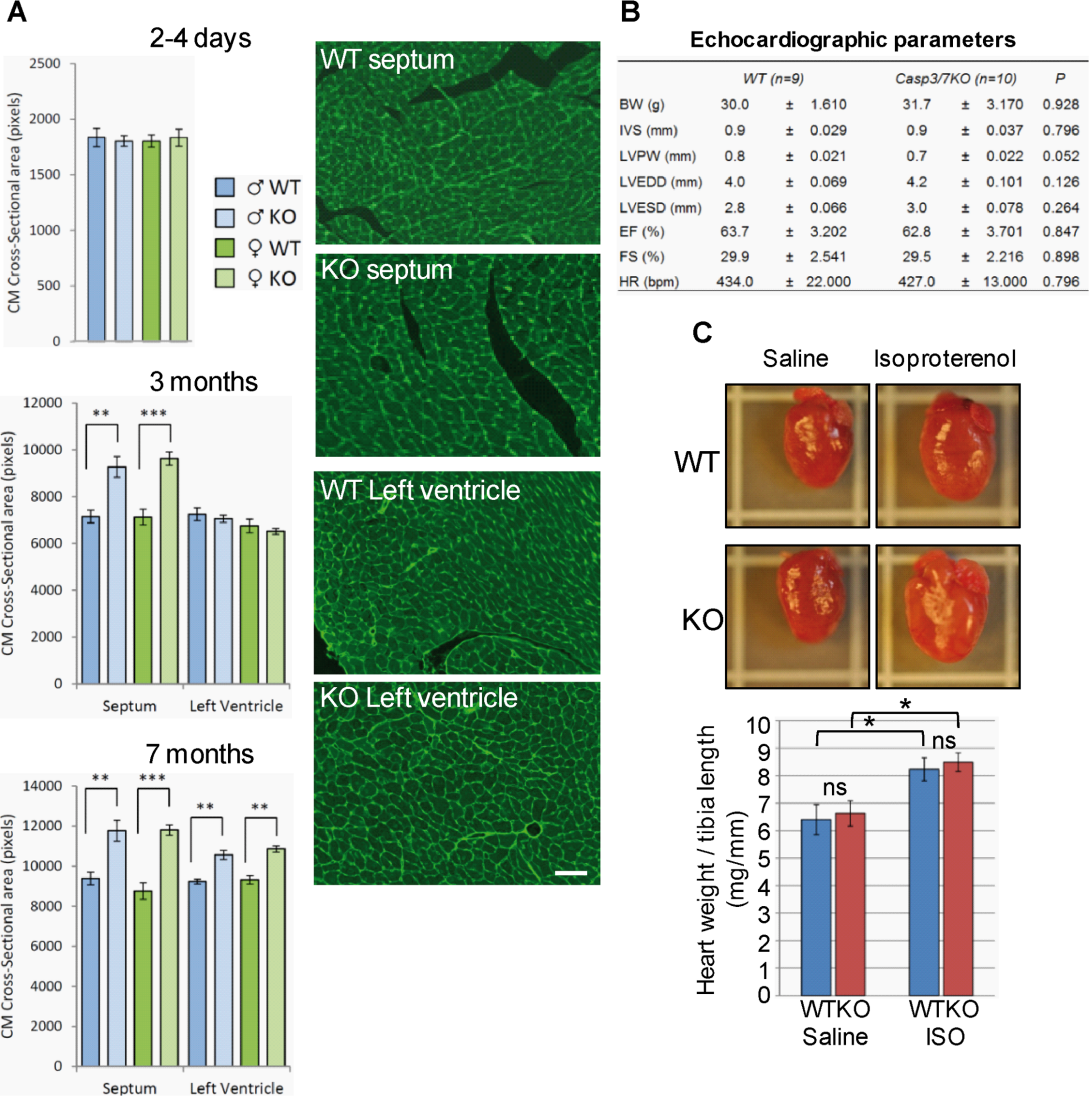
Executioner caspase-deficiency induces progressive cardiomyocyte hypertrophy without affecting heart function. **(A)** Progressive increase of cardiomyocyte (CM) cross-sectional area in the septum and ventricular wall of wild type (WT) and caspase-3 and -7 double knockout (KO) mice. **, p<0.01 KO *vs*. WT. N = 6 per gender, age and genotype. Representative microscopy images of heart histological preparations stained with FITC-WGA of 7-months-old WT and KO mice, bar: 50µm. **(B)** Cardiac function of 3-month-old wild type (WT) and caspase-3 and -7 double knockout (KO) mice. BW, body weight; IVS, inter-ventricular septum thickness; LVPW, left ventricular posterior wall thickness; LVEDD, left ventricular end diastolic diameter; LVESD, left ventricular end systolic diameter; EF, ejection fraction; FS, left ventricular fractional shortening; HR, heart rate in beats per minute. N = 9 (WT) and 10 (KO). P, statistical analysis by Student’s-t test. **(C)** Beta-adrenergic agonist-induced cardiac hypertrophy in wild type (WT) and caspase-3 and -7 double knockout (KO) mice (N = 6/genotype). Isoproterenol (ISO) at 8.8mg/kg/day or saline vehicle was administered to five 20-week-old mice per genotype using subcutaneously implanted osmotic pumps. Seven days later, heart weight and tibia length were recorded. *, p<0.05 Student’s-t test ISO *vs*. saline for each genotype. NS = not statistically significant changes. Bars are means ± s.e.m.

The increased expression of non-muscular and smooth muscle isoforms of structural genes observed by transcriptomics, and the trend towards glycolytic embryonic-like metabolism detected by proteomics in young adult knockout’s hearts were suggestive of hypertrophy. This molecular signature was associated with postnatal recovery of knockout heart’s weight in the absence of increased cardiomyocyte proliferation. Therefore, we measured cardiomyocyte cross-sectional area in cardiac slices of neonatal, young and adult wild type and double knockout mice by wheat germ agglutinin (WGA) staining, which labels extracellular matrix, as previously described [20]. Cardiomyocyte mean cross-sectional area was unaffected in neonatal hearts (Fig 4A, upper graph), but septal myocyte cross-sectional area increased by 30% in caspase-3 and-7 double knockout hearts by 3 months (Fig 4A, middle graph), and both septal and ventricular myocytes were larger in mutant hearts at 7 months (Fig 4A, lower graph and right panels). Adaptive changes in myocyte size were associated with normal heart function in adult knockout mice (Fig 4B). We next assessed whether adaptive growth of caspase-deficient myocytes was due to changes in the susceptibility of myocytes to hypertrophic stimuli. Infusion of the β-adrenergic agonist isoproterenol induced identical response in wild type and caspase knockout hearts (Fig 4C), and discarded hypersensitivity of mutant hearts to hypertrophic stimuli. The above results show that cardiomyocyte hypertrophy is a gradual response of the hypoplastic caspase-deficient heart expanding beyond the period of caspase expression in wild type hearts.

### Expression of genes involved in the control of cell proliferation in developing myocytes is regulated by executioner caspases independently of their catalytic activity

**Fig 5:**
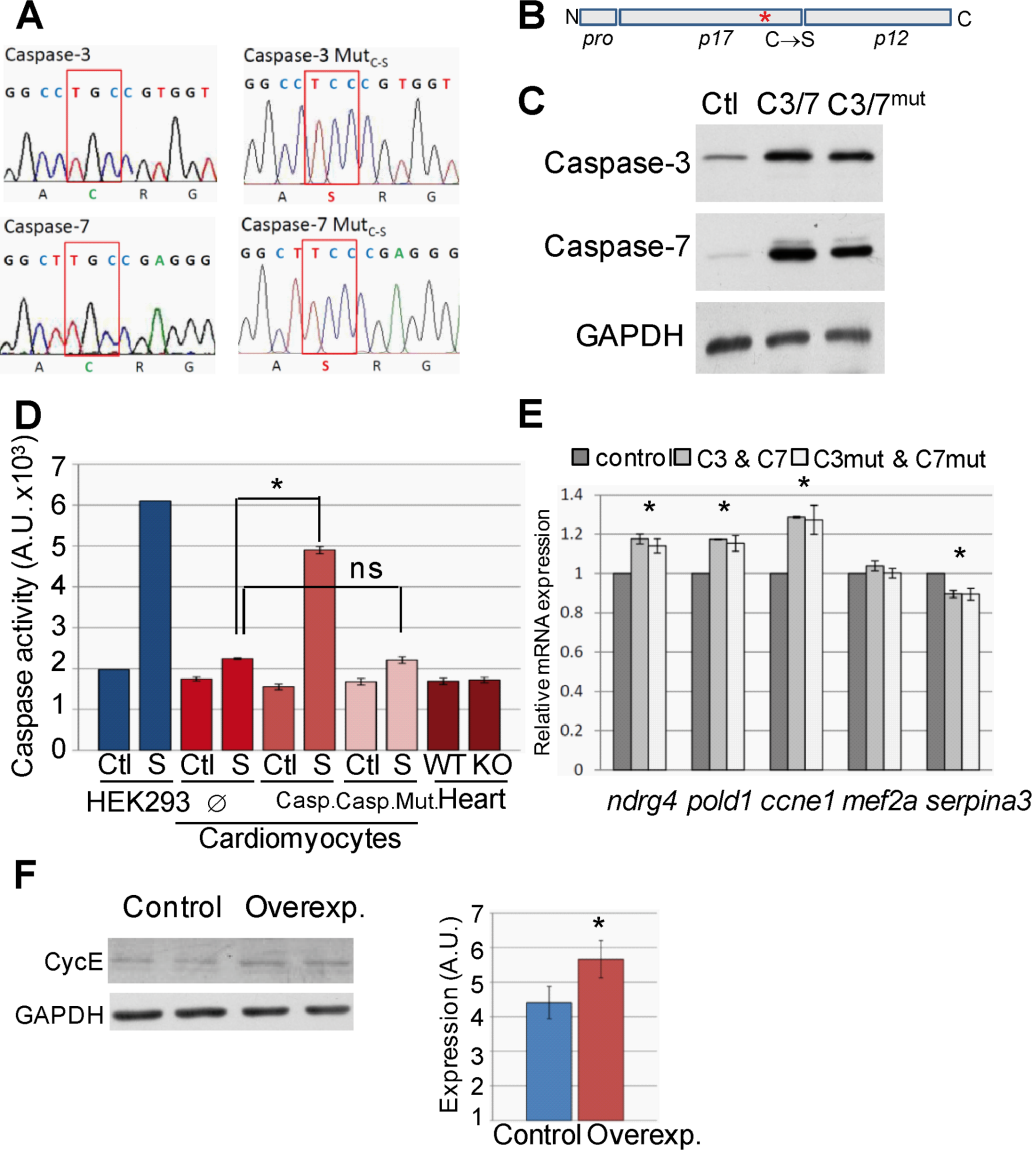
Overexpression of caspase-3 and -7 in postnatal cardiomyocytes induces increased expression of genes regulating cell division, independently of caspase proteolytic activity. **(A)** Electropherogram fragments of human Caspase-3 and Caspase-7 overexpression vectors showing the induced G→C mutation of the catalytic site’s Cystein codon that generates a Serine codon (Mut^C-S^). **(B)** Scheme of an executioner caspase showing the Cystein to Serine substitution in the p17 domain (*). N, NH_4_^+^-terminal end; C, carboxyl end. Pro: prodomain. **(C)** Overexpression efficiency of wild type (C3/7) and mutant (C3/7mut) caspases in P4-5 postnatal rat cardiomyocytes. Ctl: control with empty vector showing caspase endogenous expression. Image is representative of 3 independent experiments. **(D)** DEVDase (executioner caspase-like) enzymatic activity detected in extracts from rat neonatal cardiomyocytes overexpressing wild type (Casp.) or mutant (Casp.Mut.) executioner caspase-3 and 7 or empty vector (∅) and cultured in the absence (Ctl, control) or presence of 1 µM staurosporine (S) during 24 hours, or from neonatal wild type (WT) and caspase-3 and -7 double knockout (KO) mice’s hearts. HEK293 cell line was used as positive control of staurosporine-triggered caspase activity. *, p<0.05 and ns: not significant for comparison of staurosporine-induced caspase activity between cardiomyocytes overexpressing caspases or empty vector, Student’s-t test. N = 3. **(E)** Relative mRNA expression for different proteins whose expression is modified in caspase-3 and -7 knockout hearts was assessed in total RNA extracts from cultured P4-5 postnatal rat cardiomyocytes overexpressing wild type caspases (C3&C7), mutant caspases (C3mut&C7mut) or empty vector (control). *, p<0.05 caspase-overexpressing vs. control cultures, Student’s-t test, n = 6. **(F)** Assessment of cyclin-E expression in cardiomyocyte cultures transduced with empty (Control) or caspase-3 and -7 overexpressing vectors (Overexp.), representative blot and graph showing densitometric analysis, *, p<0.05 *vs*. control, n = 5.

To investigate how executioner caspases regulate the expression of the genes found altered by transcriptomics and proteomics in the cardiac-specific executioner caspase knockout’s heart, we overexpressed wild type caspase-3 and -7 or caspase mutants bearing a Cysteine to Serine substitution in the catalytic site in P4-5 rat postnatal myocytes (Fig 5A and 5B), which express low levels of apoptotic genes [24], and we assessed the expression of genes affected by *in vivo* caspase deletion. Rat neonatal cardiac myocytes were chose instead of mouse myocytes due to the difficulty in obtaining healthy and abundant cultures of the later and based on the validity of inter-species comparisons reported elsewhere [33]. Wild type and mutant caspases were overexpressed at similar levels (Fig 5C). Caspase proteolytic activity was altered neither in cardiomyocytes overexpressing caspase-3 and -7 nor in hearts deficient for these caspases (Fig 5D). Increased caspase activity was detected only when the kinase inhibitor staurosporine, an inducer of apoptosis [17], was added to the culture medium of the HEK293 cell line or wild type caspase-overexpressing myocytes (Fig 5D). Lack of caspase activity in normal, non-overexpressing, postnatal myocytes is due to very low expression of these genes, as previously described [24].This experiment also confirmed that Cysteine to Serine mutation abolishes caspase proteolytic activity (Fig 5D). Overexpression of wild type zymogens or the inactive mutants lead to similar increases in the expression of genes downregulated in the caspase knockout myocardium, confirming a direct implication of caspases in the control of genes involved in the regulation of myocyte proliferation (Fig 5E). We further confirmed enhanced expression at the protein level for cyclin-E (Fig 5F), which is involved in the proliferation of terminally differentiated myocytes [34]. Caspase overexpression also induced a small yet significant downregulation of serpina3 expression (Fig 5E), a gene upregulated in the caspase knockout hearts, confirming that caspase overexpression induced opposite effects than those observed in caspase-deficient myocytes. Furthermore, observation that changes in gene expression in the caspase-deficient hearts and in cardiomyocytes overexpressing either wild type or mutant caspases occur in the absence of changes in caspase activity demonstrate that gene expression regulation by executioner caspases is independent of caspase proteolytic activity.

## Discussion

The results of this study show that executioner caspase-3 and -7 are required during heart development to attain the correct number of myocytes in mice. Transcriptomic results show that caspase-3 and -7 are required for the normal expression of a broad set of genes involved in DNA replication, recombination, repair and chromatin organization as well as genes regulating cell cycle progression. Quantitative proteomics shows that executioner caspases foster the correct transition from glycolytic to oxidative metabolism in differentiating cardiomyocytes. Within the frame of the natural silencing of caspase genes during perinatal cardiac development, the above results suggest that the expression of caspases during heart development and their progressive silencing during the first weeks after birth contribute to the transition from a proliferative to a differentiated post-mitotic myocardium. In addition, we show that caspases do not require their proteolytic activity for regulating gene expression in myocytes.

Involvement of caspases in cardiomyocyte division during heart development, shown here, justify the broad expression of caspases in the myocardium during cardiac tissue growth by cell proliferation and the repression of these genes afterwards, as previously described by our group [17, 24] and others [35]. The findings presented here also improve the understanding of the involvement of executioner caspases in heart development. Previous *in vivo* studies of mice mutant for caspase-3, -7 or -8, or its positive (FADD) and negative (cFLIP) regulators, suggested the impact of caspase deficiency in heart trabeculation and ventricular wall compaction without affecting cell death [5–8]. Also, *in vitro* experiments using caspase inhibitors in cultured rodent cardiac or skeletal myocytes, P19 teratocarcinoma-derived cell line and mouse embryos showed a role of caspase activity in myocyte differentiation [10, 36]. However, phenotype rescuing by *ex vivo* culture of caspase-8 knockout embryo suggested that cardiac defects ensued secondary to placental problems in these mice, not directly to lack of caspase activity in the myocardium [11]. In addition, there are some discrepancies in the results obtained by pharmacological inhibition of caspase activity between studies performed with chicken embryos [9] and *ex-vivo* cultured mouse embryos [10]. In chicken embryos, caspase inhibitors hampered correct outflow tract looping and insertion of aortic and pulmonary arteries in the heart [9], while treatment of E12.5 mouse embryos with caspase inhibitors had no impact on the formation of these structures [10] despite the ongoing construction of the arterial connections at this period of mouse embryo development [37]. Here, we show that the most evident phenotype of the *Nkx2*.*5*::*Cre*-driven conditional caspase-3 null / caspase-7 null mouse is reduced cardiomyocyte number and this effect seems independent to proteolytic activity since no caspase-like activity was detected in the myocardium and the effects on gene expression of caspase overexpression in neonatal myocytes occurred also in the absence of increased caspase activation. Therefore, *in vivo* results suggest that cardiac phenotypes previously described of mice with ubiquitous caspase deletion and changes in chicken embryos and *ex-vivo* cultured mouse embryos treated with caspase inhibitors could probably be due to inhibition of other enzymes or blockade of caspase activity in other cells or embryo tissues, not related to caspase function specifically in cardiomyocytes. Alternatively, embryos with cardiac-specific deletion of caspases could activate cardiac adaptive responses to circumvent limited differentiation but cannot completely recover cardiomyocyte number.

Inhibition of Wnt/β-catenin signaling contributes to heart formation [38], while Wnt signaling allows cardiomyocyte expansion [39, 40]. Wnt11 promotes cardiac differentiation [41] and a peak of caspase-3/7-like activity induced by Wnt11 was described to be required for P19 carcinoma cell line differentiation to myocytes (e.g. expression of *troponin T* _*tnt*_) at least in part through β-catenin degradation [10]. This was in agreement with progressive reduction of Wnt activity in the developing mouse myocardium. In addition, Wnt signaling was blunted in the heart by caspase inhibitors in *ex-vivo* cultured E12.5 and E14.5 embryos resulting in reduced number of α-myosine heavy chain-positive (MHC^+^) cardiomyocytes detected by immunofluoerscence [10]. However, we did not detect changes in MHC expression by *in vivo* deletion of executioner caspase-3 and -7 in cardiomyocytes in neonatal hearts. Lack of change in MHC expression despite reduced cardiomyocyte number can be explained probably because most of the myocardial protein comes from myocytes and therefore, reduced myocyte number does not affect relative structural protein abundance (i.e. fibroblast’s protein contributes minimally to the total protein of the myocardium). To gain further insight into the potential relationship between caspases and Wnt signaling in our model, we assessed β-catenin expression in protein extracts of neonatal and young hearts from wild type and caspase-deficient mice and of rat neonatal cardiomyocytes overexpressing caspase-3 and 7. Although β-catenin expression was reduced with age, we show that it was modified by executioner caspase abundance neither *in vivo* nor *in vitro* (Figure F in S3 File). These results could refine previous reports suggesting that caspase-3 target β-catenin and showing that other proteases could be involved in β-catenin processing [10].

The prevalence of glycolysis in neonatal caspase deficient hearts, as indicated by the proteome analysis of newborn samples, suggests an initial retardation of the switch from glycolytic to oxidative metabolism occurring through normal heart maturation [32]. Growing evidence indicates that metabolic status influences or is influenced by proliferation [42]. In fact, reduced mitochondrial activity blocks proliferation without compromising cell growth in hepatocytes [43] and cyclinB1/cdk1-induced phosphorylation of mitochondrial proteins improves mitochondrial respiration, facilitating cell proliferation [44]. Our results show that caspase-3 is expressed in mitochondria and cytosol, in agreement with previous reports [45], whereas caspase-7 is mostly cytosolic. They also show that caspase-3 compensates for caspase-7 deficiency in the regulation of gene expression, but the reverse is only partial. Together, these findings suggest a relevant function of caspase-3 in mitochondria that could be involved in the control of genes regulating cell division. Based in the above facts, we speculate that a reduction in the expression of the machinery for mitochondrial oxidative metabolism induced by caspase deficiency in cardiomyocytes could compromise proliferation but not growth.

A limitation of our study is intrinsic to the *in vivo* approach, which is, in turn, one of its stronger points respect previous works. Live models developed in their natural environment have a powerful capacity to adapt for surviving and hence, vital functions of the targeted genes can be carried out through alternative pathways, softening the phenotype. This is the case of the E2F1-null and E2F1/E2F2 compound-mutant mice, which develop and reproduce normally [24, 46, 47] yet show abnormal T lymphocyte maturation and pancreatic dysfunction respectively, despite the relevant role of E2F1 and E2F2 in many cellular functions including cell division [48]. Therefore, cardiac-specific caspase-3 null / caspase-7 null mouse’s hypoplastic phenotype could ensue from lack of their most irreplaceable function during heart development. Previous studies of chicken embryos and *ex vivo* cultured mouse embryos treated with caspase inhibitors found altered cardiac formation and lack of myocyte differentiation as measured by sarcomeric protein expression that could be due to drugs targeting other cells or tissues or even other enzymes [9, 10]. Nevertheless, our study cannot discard a role of caspases in the signaling for cardiomyocyte structural differentiation, which could be accomplished by alternative pathways in the cardiomyocyte-specific executioner caspase-deficient mouse.

Finally, our results suggest that the function of caspase-3 and -7 in developing cardiomyocytes is independent of their proteolytic activity. Prodomain-mediated signaling could account for proteolytic-independent functions of caspases with long prodomain such as caspase-2 and -8 [49], but the short prodomain of executioner caspases challenge the validity of this hypothesis for the cardiac function of caspase-3 and -7 reported here. However, catalytic-independent role of caspase-3 in fibronectin secretion by fibroblasts has been recently reported [50]. Thus, although we do not provide a molecular mechanism by which caspases regulate gene expression, our results suggest that non-proteolytic activity of executioner caspases is more common than assumed.

Transcriptomic comparison of wild type and executioner caspase-deficient hearts at different developmental time points underscored the influence of executioner caspases on the expression of genes regulating different aspects of cell division, such as DNA replication, chromosome segregation and cell cycle progression. Quantitative proteomics allowing simultaneous comparison of four time-points identified changes in metabolic pathways showing reduced abundance of proteins involved in oxidative phosphorylation and increased glycolytic protein abundance in neonatal knockout hearts compared to wild types, which was progressively normalized during postnatal development. Thus, combining transcriptomics, which were later confirmed by protein electrophoresis and Western Blot, and proteomics contributed information on complementary molecular signaling altered by the lack of executioner caspases during heart development. The report of non-canonical role of executioner caspases in the regulation of cell proliferation, maturation and growth, irrespective of their proteolytic activity and through the regulation of gene expression to influence heart development, opens an exciting field of biomedical research that could contribute clues to understand and eventually treat cardiac congenital diseases.

## Conclusions

The work presented here shows that: 1) Executioner caspase-3 and 7 are expressed in the developing rodent heart and are progressively silenced after birth. 2) Subcellular distribution of these caspases differs: caspase-3 is expressed in mitochondria and cytosol and caspase-7 is mostly cytosolic. 3) Lack of caspase-3 in cardiomyocytes accompanied by ubiquitous deletion of caspase-7 during mouse development reduces the final number of cardiomyocytes. 4) The hypoplastic neonatal heart of caspase-3 and 7 double null mouse express abnormally low levels of genes regulating DNA replication/repair and cell cycle and express abnormally high levels of non-myocyte sarcomeric genes that usually are repressed during differentiation. 5) The proteomic profile of the neonatal caspase-deficient myocardium shows abnormally high abundance of glycolytic enzymes and lower abundance of proteins involved in oxidative phosphorylation compared to wild type hearts. 6) These changes in gene expression *in vivo* are not correlated with changes in caspase-like activity and proteolytically inactive caspases overexpressed in neonatal myocytes can affect the expression of some genes regulating cell division with identical efficiency than wild type caspases.

## Supporting Information

S1 File The ARRIVE Guidelines checklist for reporting animal studies. (PDF)

S2 File Supporting methodological information: Microarray procedures, Proteomics procedures,genotyping primers and antibody specifications. (PDF)

S3 File Supporting figures. Figure A. Intercross design to obtain caspase-3 and 7 double knockouts, Figure B. Genotyping, Figure C. Array results of several gene families including genes shown in Fig 2B, Figure D. Expression in single knockout and Cre-overexpressing hearts of key genes differentially expressed in the caspase-3 and 7 double knockout hearts, Figure E. Statistical analysis of quantitative proteomics data, Figure F. Beta-catenin expression in wild type and Caspase-3 and 7 knockout hearts and in neonatal cardiomyocytes overexpressing caspases, Figure G. Identification of the proteins presented in Fig 3B (OxPhos protein set).
(PDF)
