# Supplementary material for: Executioner Caspase-3 and 7 Deficiency Reduces Myocyte Number in the Developing Mouse Heart
Source: PLoS One. 2015 Jun 29;10(6):e0131411. doi: 10.1371/journal.pone.0131411 (PMC4487935; doi:10.1371/journal.pone.0131411)
Supplement: S2 File — (PDF) [file pone.0131411.s002.pdf]

S2 File. Supporting information.

Experimental Procedures.

Expression microarrays RNA was extracted from either six wild-type (three males and three females) or double knockout hearts at P0-1 and at P30-35, totaling 24 samples. Minimal experimental size for robust statistical meaning was calculated in a previous microarray test and set at 5 animals per group (available upon request). All heart RNA was from ventricle tissue only and passed several quality controls as scheduled by the facility. Microarray analysis was performed at the Microarray Core Facility of the Vall d'Hebron Institute of Research (VHIR, Barcelona, Spain) High Technology Unit using 24 Genechip Mouse 1.0 ST arrays processed on a Gene Titan System (Affymetrix). Starting material was 100 ng of total RNA from ventricular samples. Quality of isolated RNA was first measured by Bioanalyzer Assay (Agilent). Sense ssDNA was fragmented, labelled and hybridized to the arrays. Chips were processed on an Affymetrix GeneChip Fluidics Station 450 and Scanner 30 00. All microarray data derived from our study is deposited in NCBI's Gene Expression Omnibus and are accessible through GEO Series accession number GSE63023.
Bioinformatic analysis was performed at the Statistics and Bioinformatics Unit (UEB) of the VHIR. Robust Multi-array Average (RMA) algorithm [1] was used for pre-processing microarray data. Background adjustment, normalization and summarization of raw core probe expression values were defined so that the exon level values were averaged to yield one expression value per gene. The analysis was done considering time and genotype factors. Data were subjected to non-specific filtering to remove low signal and low variability genes. Conservative (low) thresholds were used to reduce possible false negative results. This yielded a list of 6599 genes to be analyzed. Selection of differentially expressed genes was based on a linear model analysis with empirical Bayes modification for the variance estimates [2]. To account for multiple testing, P-values were adjusted to obtain stronger control over the false discovery rate (FDR), as described by the Benjamini and Hockberg method. The analysis of biological significance was based on Gene Ontology (GO) and KEGG enrichment analyses, and was performed on those genes with unadjusted P-value below 0.01 in all cases. In addition to that, in order to detect and overlap the most significant pathways across the resulting sets of genes, QIAGEN's Ingenuity Pathway Analysis (IPA®, QIAGEN) was performed using distinct data filtering criteria: from a log fold change cut-off of 0.5 to a log fold change of 2.0 combined with FDR at 0.05, depending on the contrast.

Proteomics data collection and iTRAQ quantitative analysis Ventricle samples from 1-day-old neonate, 1-month, 3-month and 8-month-old mice were analyzed. A pool of two independent samples per age and genotype were pooled. Proteins were digested using the filter aided sample preparation (FASP) protocol [3]. Briefly, samples were dissolved in 50 mM Tris-HCl pH8.5, 4% SDS and 50 mM DTT, boiled for 10 min and centrifuged. Protein concentration in the supernatant was measured by the Direct Detect® Spectrometer (Millipore). About 150 μg of protein were diluted in 8 M urea in 0.1 M Tris-HCl (pH 8.5) (UA), and loaded onto 30 kDa centrifugal filter devices (FASP Protein Digestion Kit, Expedeon, TN, USA). The denaturation buffer was replaced by washing three times with UA. Proteins were then alkylated using 50 mM iodoacetamide in UA for 20 min in the dark, and the excess of alkylation reagents was eliminated by washing three times with UA and three additional times with 50 mM ammonium

bicarbonate. Proteins were digested overnight at 37ºC with modified trypsin (Promega, Madison, WI, USA) in 50 mM ammonium bicarbonate at 40:1 protein:trypsin (w/w) ratio. The resulting peptides were eluted by centrifugation with 50 mM ammonium bicarbonate (twice) and 0.5M sodium chloride. Trifluoroacetic acid (TFA) was added to a final concentration of 1% and the peptides were finally desalted onto C18 Oasis-HLB cartridges and dried-down for further analysis.

For stable isobaric labeling, the resulting tryptic peptides were dissolved in Triethylammonium bicarbonate (TEAB) buffer, and the concentration of peptides was determined by measuring amide bonds with the Direct Detect system. Equal amounts of each peptide sample were labeled using the 8-plex iTRAQ Reagents Multiplex Kit (Applied Biosystems, Foster City, CA, USA) according to manufacturer's protocol. Briefly, each peptide solution was independently labeled at room temperature for 1 h with one iTRAQ reagent vial (mass tag 113, 114, 115, 116, 117, 118, 119 or 121) previously reconstituted with isopropanol. After incubation at room temperature for 2 h, reaction was stopped with diluted TFA and peptides were combined. Samples were concentrated in a Speed Vac, desalted onto C18 Oasis-HLB cartridges and dried-down for further analysis as previously described.

Labeled peptides were loaded into the LC-MS/MS system for on-line desalting onto C18 cartridges and analyzing by LC-MS/MS using a C-18 reversed phase nano-column (75 µm I.D. x 50 cm, 2 µm particle size, Acclaim PepMap RSLC, 100 C18; Thermo Fisher Scientific, Waltham, MA, USA) in a continuous acetonitrile gradient consisting of 0-30% B in 360 min, 50-90% B in 3 min (A= 0.5% formic acid; B=90% acetonitrile, 0.5% formic acid). A flow rate of 200 nl/min was used to elute peptides from the RP nano-column to an emitter nanospray needle for real time ionization and peptide fragmentation on a Q-Exactive mass spectrometer (Thermo Fisher). An enhanced FT-resolution spectrum (resolution=70.000) followed by the MS/MS spectra from the 15 most intense parent ions were analyzed along the chromatographic run. Dynamic exclusion was set at 30 s. For increasing proteome coverage, iTRAQ-labeled samples were also fractionated by cation exchange chromatography (Oasis HLB-MCX columns) into six fractions, which were desalted and analyzed by using the same system and conditions described before.

For peptide identification, all spectra were analyzed with Proteome Discoverer (version 1.4.0.29, Thermo Fisher Scientific) using SEQUEST-HT (Thermo Fisher Scientific). For database searching at the Uniprot database containing all sequences from mouse (March 03, 2013), parameters were selected as follows: trypsin digestion with 2 maximum missed cleavage sites, precursor and fragment mass tolerances of 2 Da and 0.02 Da, respectively, carbamidomethyl cysteine as fixed modification and methionine oxidation as dynamic modifications. For iTRAQ labeled peptides, N-terminal and Lys iTRAQ modifications were selected as a fixed modification. Peptide identification was validated using the probability ratio method [4] with an additional filtering for precursor mass tolerance of 12 ppm. False discovery rate (FDR) was calculated using inverted databases and the refined method [5] was used to filter peptides for quantitation, as previously described [6]. Protein quantification from reporter ion intensities and statistical analysis of quantitative data were performed using QuiXoT, based on a statistical model previously described [5, 7]. In this model protein log2-ratios are expressed in form of the standardized variables, i.e., in units of standard deviation according to their estimated variances (Zq values). For functional protein analysis,

proteins were classified in terms of the Gene Ontology Biological Process and Cellular Component, KEGG, IPA, and DAVID and analysis of altered categories was analyzed as described [7]. Alterations in the abundance of functional categories were visualized by comparing the cumulative frequency (sigmoid) plots of the standardized variable with that of the normal distribution, as in previous works [8-10]. All proteomic data derived from our study is deposited in Peptide Atlas and are accessible through the accession number PASS00689.

Genotyping* primer sequences.

Primer	Sequence 5'-3'
Cre *Fwd*	AGG TTC GTT CAC TCA TGG A
Cre *Rev*	TCG ACC AGT TTA GTT ACC C
Caspase 3 *H*	GAG CCT TCA TAG GGG TGC AA
Caspase 3 *J*	GGG GAG CAG AGG GAA TAA AG
Caspase 3 *K*	CAT AGA ATC CCA AGC CAG GA
Caspase 7 WT *Fwd*	GAC TGC TTC CAC AGC CTC TAA CTG
Caspase 7 WT *Rev*	GTC TGG TAA AGT GCG GAG AAC G
Caspase 7 Neo *Fwd*	ATC CTT TAT GGG TGT CAC GCC
Caspase 7 Neo *Rev*	TGC TAA AGC GCA TGC TCC AGA CTG

*Genotyping PCR conditions will made available upon request. Cre: 250 bp. Caspase3: WT allele: ± 180 bp Floxed allele: ± 230 bp Deleted allele: ± 320 bp. Caspase-7: WT allele: ± 240 bp KO allele: ± 310 bp

Antibody specifications.

Antigen	Supplier	Dilution
α- actin	Sigma-Aldrich (A2172)	1/5,000
Caspase 3	Cell Signaling (9662)	1/3,000
Caspase 6	Cell Signaling (9762)	1/1,000
Caspase 7	Enzo Life Sciences (ADI-AMM-127)	1/1,000
Cenpa	Abcam (ab33565)	1/1,000
Cdc6	Santa Cruz Biotechnology (sc-9964)	1/200
Ccne1	Cell Signaling (4129)	1/1,000
Dihidrolipoil Dehydrogenase (DLD)	Abcam (ab133551)	1/20,000
DNA Polymerase Delta (PolD1)	Abcam (ab10362)	1/1,000
Gapdh	Abcam (ab8245)	1/10,000
Glut4	Abcam (ab33780)	1/1,000
Lamin A+C	Abcam (ab8984)	1/1,000
Mef2a	Cell Signaling (9736)	½,000
Ndrg4	Cell Signaling (9039)	1/1,000
Serpina3	Sigma-Aldrich (HPA002560)	1/250

References for Supporting information. Experimental procedures

1. Irizarry RA, Hobbs B, Collin F, Beazer-Barclay YD, Antonellis KJ, Scherf U, et al. Exploration, normalization, and summaries of high density oligonucleotide array probe level data. Biostatistics. 2003;4(2):249-64. doi: 10.1093/biostatistics/4.2.249. PubMed PMID: 12925520.
2. Smyth GK. Linear models and empirical bayes methods for assessing differential expression in microarray experiments. Stat Appl Genet Mol Biol. 2004;3:Article3. doi: 10.2202/1544-6115.1027. PubMed PMID: 16646809.
3. Wiśniewski JR, Zougman A, Nagaraj N, Mann M. Universal sample preparation method for proteome analysis. Nat Methods. 2009;6(5):359-62. doi: 10.1038/nmeth.1322. PubMed PMID: 19377485.
4. Martínez-Bartolomé S, Navarro P, Martín-Maroto F, López-Ferrer D, Ramos-Fernández A, Villar M, et al. Properties of average score distributions of SEQUEST: the probability ratio method. Mol Cell Proteomics. 2008;7(6):1135-45. doi: 10.1074/mcp.M700239-MCP200. PubMed PMID: 18303013.
5. Navarro P, Trevisan-Herraz M, Bonzon-Kulichenko E, Núñez E, Martínez-Acedo P, Pérez-Hernández D, et al. General statistical framework for quantitative proteomics by stable isotope labeling. J Proteome Res. 2014;13(3):1234-47. doi: 10.1021/pr4006958. PubMed PMID: 24512137.
6. Bonzon-Kulichenko E, Pérez-Hernández D, Núñez E, Martínez-Acedo P, Navarro P, Trevisan-Herraz M, et al. A robust method for quantitative high-throughput analysis of proteomes by 18O labeling. Mol Cell Proteomics. 2011;10(1):M110.003335. doi: 10.1074/mcp.M110.003335. PubMed PMID: 20807836; PubMed Central PMCID: PMCPMC3013457.
7. Jorge I, Burillo E, Mesa R, Baila-Rueda L, Moreno M, Trevisan-Herraz M, et al. The human HDL proteome displays high inter-individual variability and is altered dynamically in response to angioplasty-induced atheroma plaque rupture. J Proteomics. 2014;106:61-73. doi: 10.1016/j.jprot.2014.04.010. PubMed PMID: 24747125.
8. Bonzon-Kulichenko E, Martínez-Martínez S, Trevisan-Herraz M, Navarro P, Redondo JM, Vázquez J. Quantitative in-depth analysis of the dynamic secretome of activated Jurkat T-cells. J Proteomics. 2011;75(2):561-71. doi: 10.1016/j.jprot.2011.08.022. PubMed PMID: 21920478.
9. Isern J, Martín-Antonio B, Ghazanfari R, Martín AM, López JA, del Toro R, et al. Self-renewing human bone marrow mesenspheres promote hematopoietic stem cell expansion. Cell Rep. 2013;3(5):1714-24. doi: 10.1016/j.celrep.2013.03.041. PubMed PMID: 23623496.
10. Martínez-Acedo P, Núñez E, Gómez FJ, Moreno M, Ramos E, Izquierdo-Álvarez A, et al. A novel strategy for global analysis of the dynamic thiol redox proteome. Mol Cell Proteomics. 2012;11(9):800-13. doi: 10.1074/mcp.M111.016469. PubMed PMID: 22647871; PubMed Central PMCID: PMCPMC3434769.
