# Supplementary material for: Executioner Caspase-3 and 7 Deficiency Reduces Myocyte Number in the Developing Mouse Heart
Source: PLoS One. 2015 Jun 29;10(6):e0131411. doi: 10.1371/journal.pone.0131411 (PMC4487935; doi:10.1371/journal.pone.0131411)
Supplement: S3 File — Figure A. Intercross design to obtain caspase-3 and 7 double knockouts, Figure B. Genotyping, Figure C. Array results of several gene families including genes shown in Fig 2B, Figure D. Expression in single knockout and Cre-overexpressing hearts of key genes differentially expressed in the caspase-3 and 7 double knockout hearts, Figure E. Statistical analysis of quantitative proteomics data, Figure F. Beta-catenin expression in wild type and Caspase-3 and 7 knockout hearts and in neonatal cardiomyocytes overexpressing caspases, Figure G. Identification of the proteins presented in Fig 3B (OxPhos protein set). (PDF) [file pone.0131411.s003.pdf]

Figure A

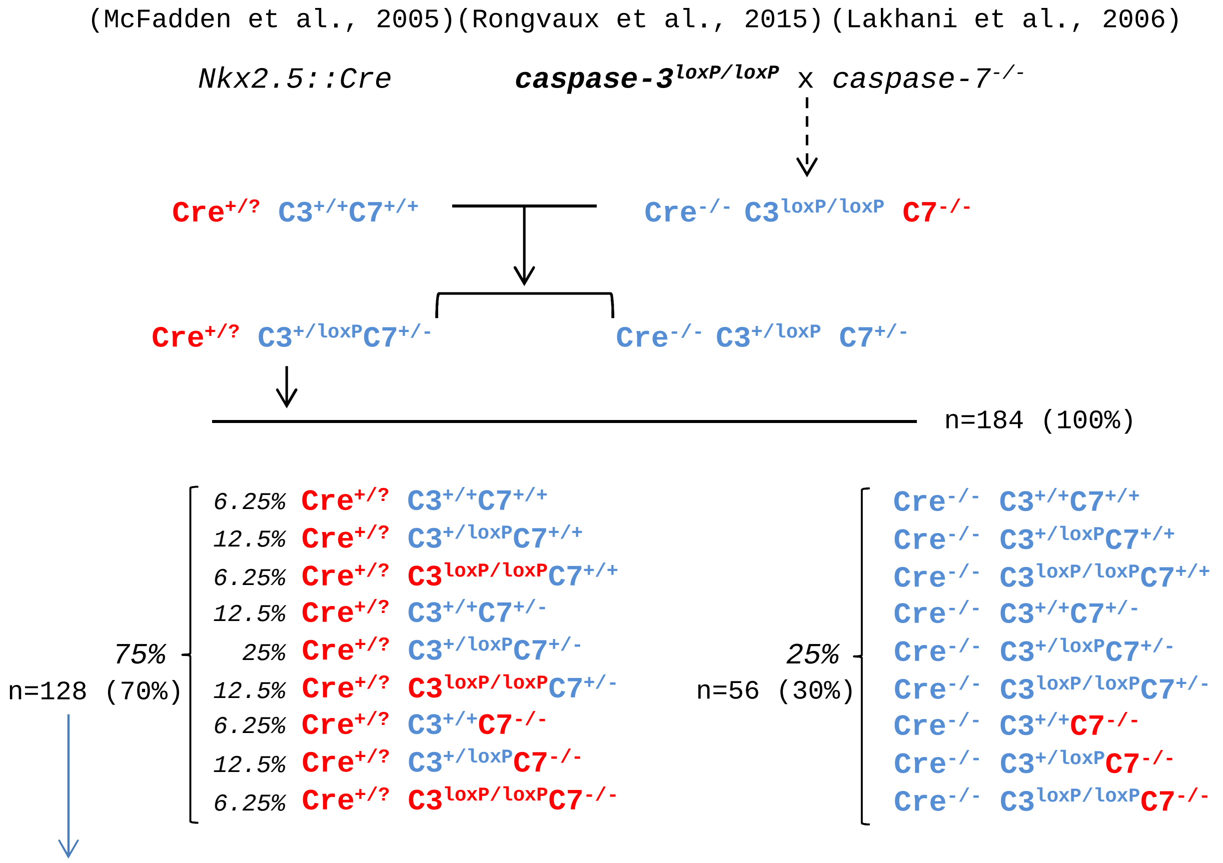

Genotype	n	% of Cre+ (75%)	% of total (100%)	
$C3^{+/loxP}$ $C7^{+/-}$	36	28%	19.6%	
$C3^{+/loxP}$ $C7^{+/+}$	13	10.1%	7.07%	
$C3^{+/loxP}$ $C7^{-/-}$	17	13.2%	9.24%	
$C3^{+/+}C7^{+/-}$	15	11.7%	8.19%	
$C3^{loxP/loxP}$ $C7^{+/-}$	17	13.2%	9.24%	
$C3^{+/+}$ $C7^{+/+}$	7	5.4%	3.78%	Wild type
$C3^{+/+}$ $C7^{-/-}$	6	4.7%	3.29%	Caspase-7 KO
$C3^{loxP/loxP}$ $C7^{+/+}$	8	6.25%	4.37%	Caspase-3 KO
$C3^{loxP/loxP}$ $C7^{-/-}$	9	7%	4.9%	Double KO

Intercross design to obtain wild type, caspase-3KO, caspase-7KO and double mutant mice. Caspase-3 floxed mice were generated as described in Rongvaux et al., 2015 and crossed with caspase-7KO mice (Lakhani et al., 2006) and their *caspase-3*$^{loxP/loxP}$ *caspase-7*$^{-/-}$ descendants were crossed with line *Nkx2.5::Cre* (McFadden et al., 2005). *Nkx2.5::Cre; caspase-3*$^{+/loxP}$*; caspase-7*$^{+/-}$ mice were intercrossed to obtain the genotypes described in the figure. Expected (Mendelian) ratios are shown in italics and actual ratios are shown in parenthesis and in the table. N is the number of animals in each group included for calculations.

Figure B

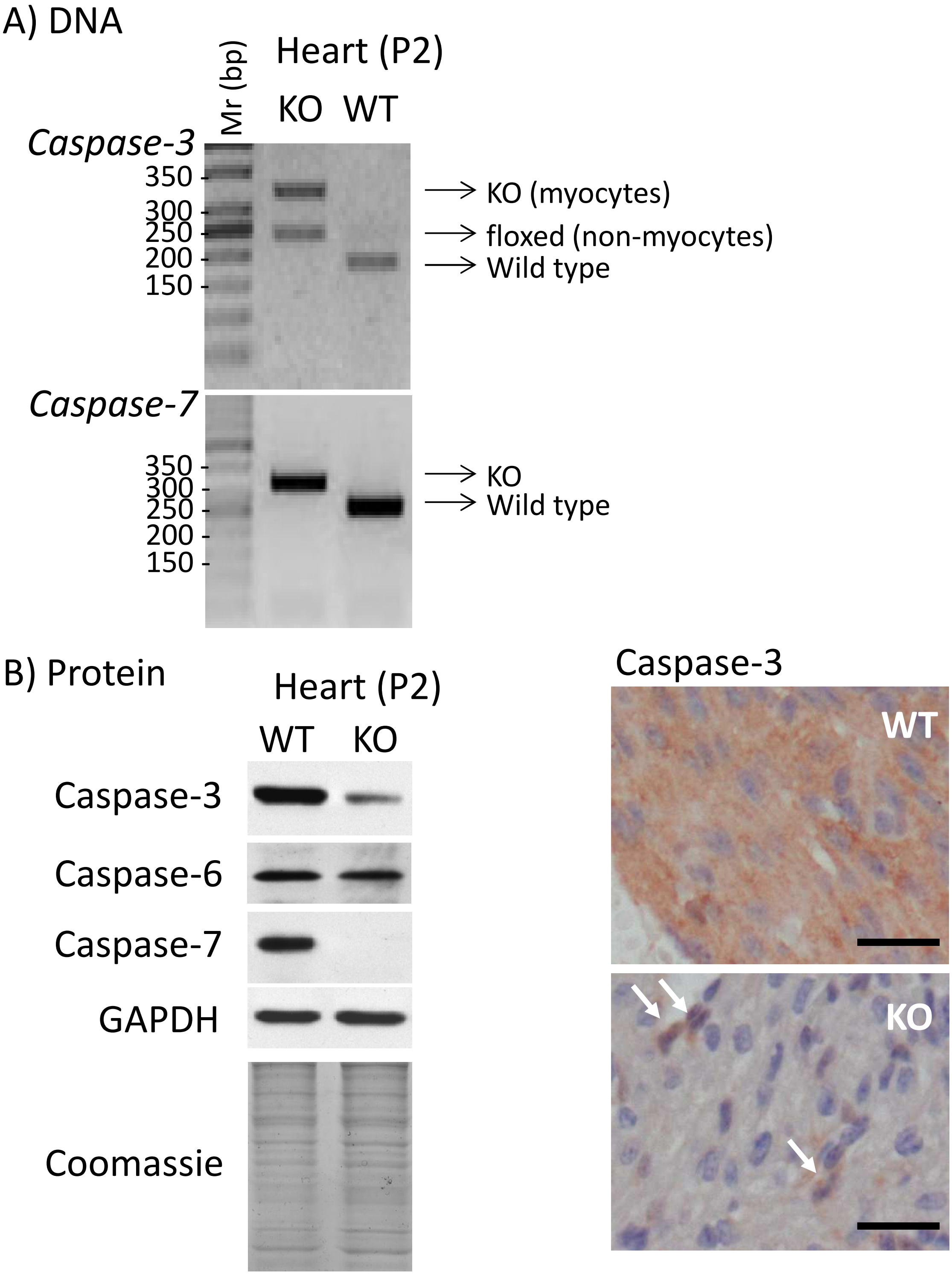

Genotyping the hearts of wild type and Nkx2.5-driven caspase-3KO/caspase-7KO mice. A) Genomic PCR. Primers and amplicon sizes are described in S1 Table. DNA was extracted as described in Materials and Methods section from P2 neonatal hearts and PCR was performed (protocol will be sent upon request). The KO sample shows KO band due to myocyte DNA and floxed band due to non-myocytes. B) Protein detection by Western Blot. Caspase-3, Caspase-6 and Caspase-7 and GAPDH antibody specifications are shown in S2 Table. Caspase-3 band in the KO heart is expressed by non-myocytes (cardiac fibroblasts, endothelial cells, etc) as observed in the caspase-3 immunohistochemistry shown in the right panels, using the same antibody as in the Western Blot. Arrows in the KO image indicate non-myocytes (narrow nucleus, perivascular). Band: 25µm.

Figure C

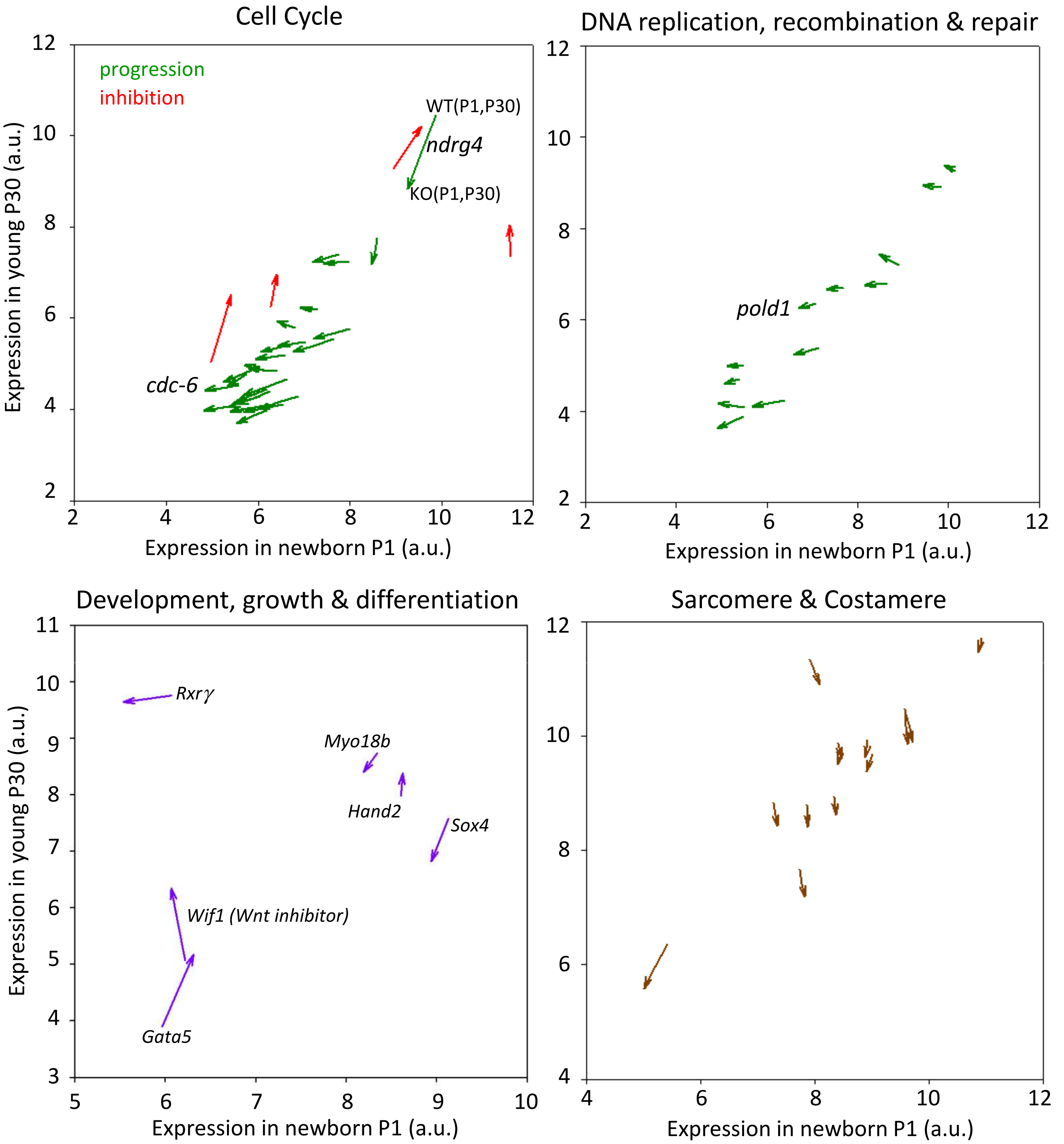

Trend of gene expression changes comparing newborn and young values. Each arrow links the wild type (WT) data with the caspase-3 and 7KO data for the expression values (as indicated in an example in the top left graph) of a selection of the genes showing the most statistically significant changes (expression values from the array). Arrows pointing to the left indicate lower expression in KO vs. WT newborns with no changes due to genotype in the young. Arrows pointing to the bottom indicate lower expression in the young KO vs. WT with no changes due to genotype in the newborns. Arrows pointing to the top indicate higher values in the young KO vs. WT with no changes due to genotype in newborns. Arrows pointing to the right indicate higher expression in the newborn KO vs. WT.

Figure D

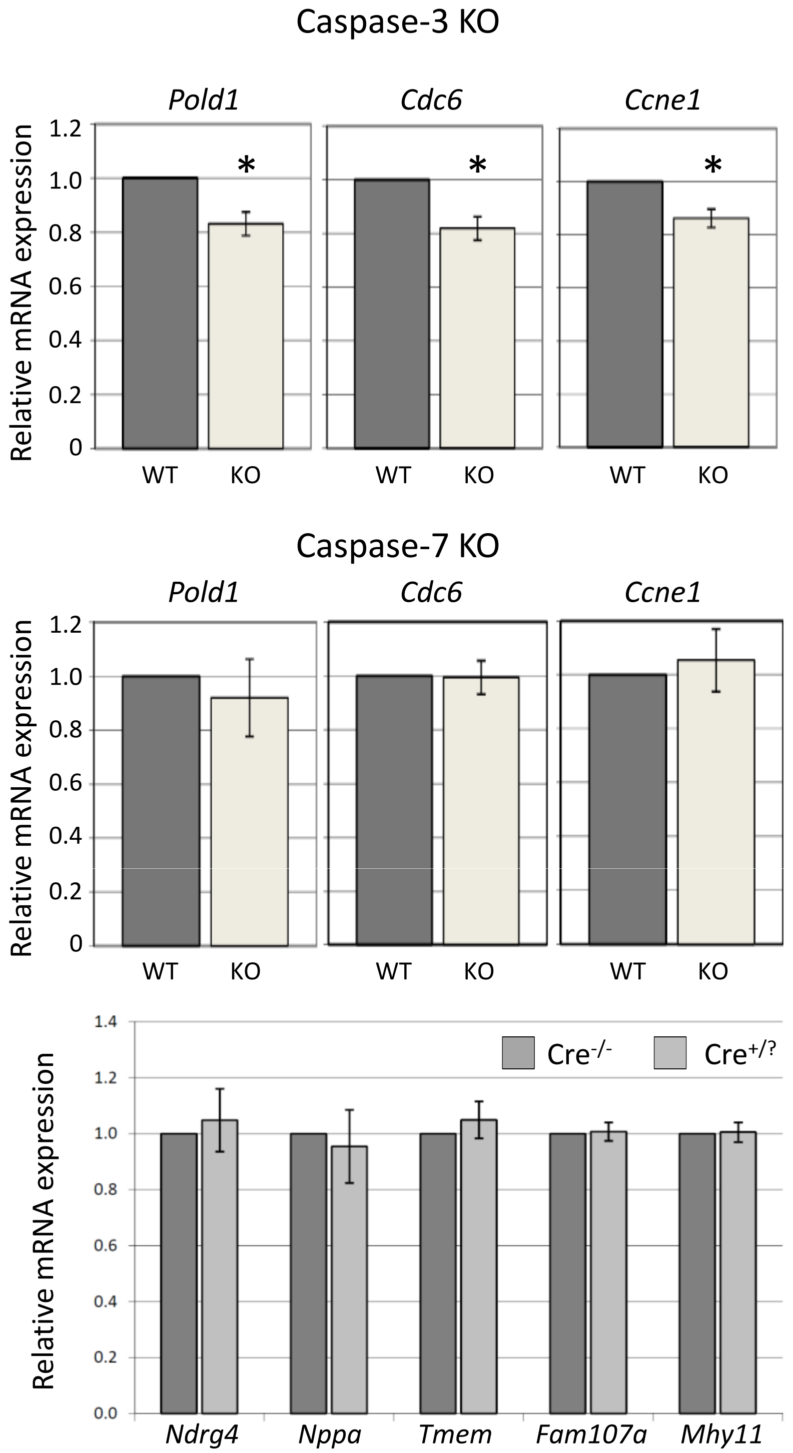

Effect of cardiac-specific caspase-3 deletion, caspase-7 deletion or Cre recombinase expression in the cardiac expression of genes affected by caspase-3 and caspase-7 double deletion (Fig. 3). Gene expression was analyzed by qPCR in neonatal heart extracts of wild type (WT), caspase-3 or caspase-7 single knockout mice (Fig. S1), or young Nkx2.5::Cre mice (expressing Cre or not, $Cre^{+/?}$, $Cre^{-/-}$, respectively), to assess changes in the abundance of mRNA of genes regulated by caspases-3 and 7 double deletion (Fig. 3). N=5, * p<0.05 vs. WT.

Figure E

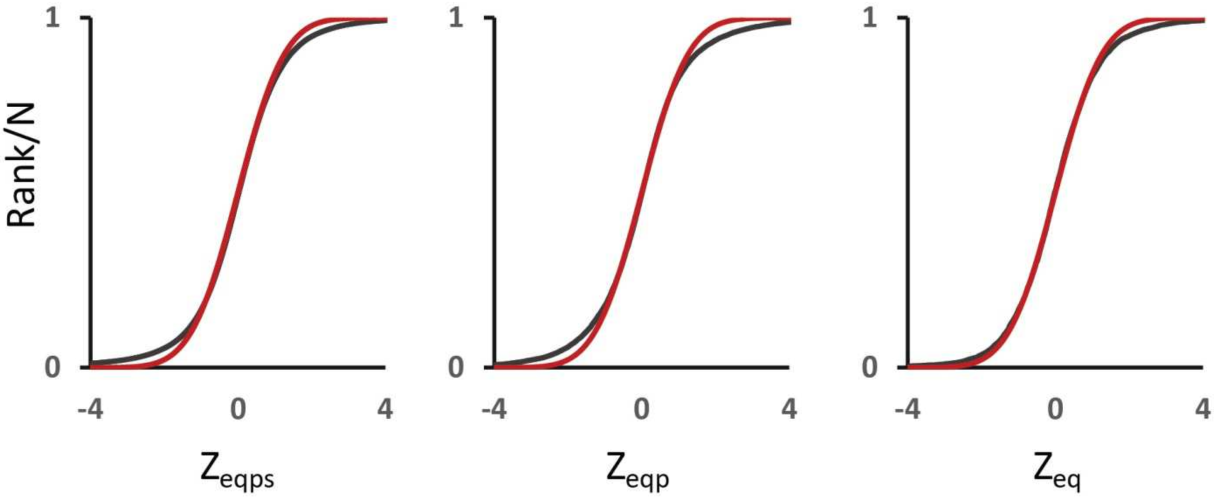

Statistical analysis of quantitative proteomics data.- The mass spectrometry data was analysed using the WSPP model developed in our laboratory, as described in Supplemental Information. To show the accuracy of the model for the analysis of the data at the spectrum, peptide and protein levels, sigmoid plots were constructed showing the cumulative distributions of log2-ratios in units of the standard deviation at each level (**Zeqps**, spectrum to peptide variability; **Zeqp**, peptide to protein variability; **Zeq**, protein to grand mean variability). Red lines are drawn according to the theoretical normal distribution with zero mean and unit variance. For clarity, only one of the several possible comparisons is shown (tags 113 and 117; WT vs. KO in neonate); similar results were obtained with the rest of comparisons (not shown).

Figure F

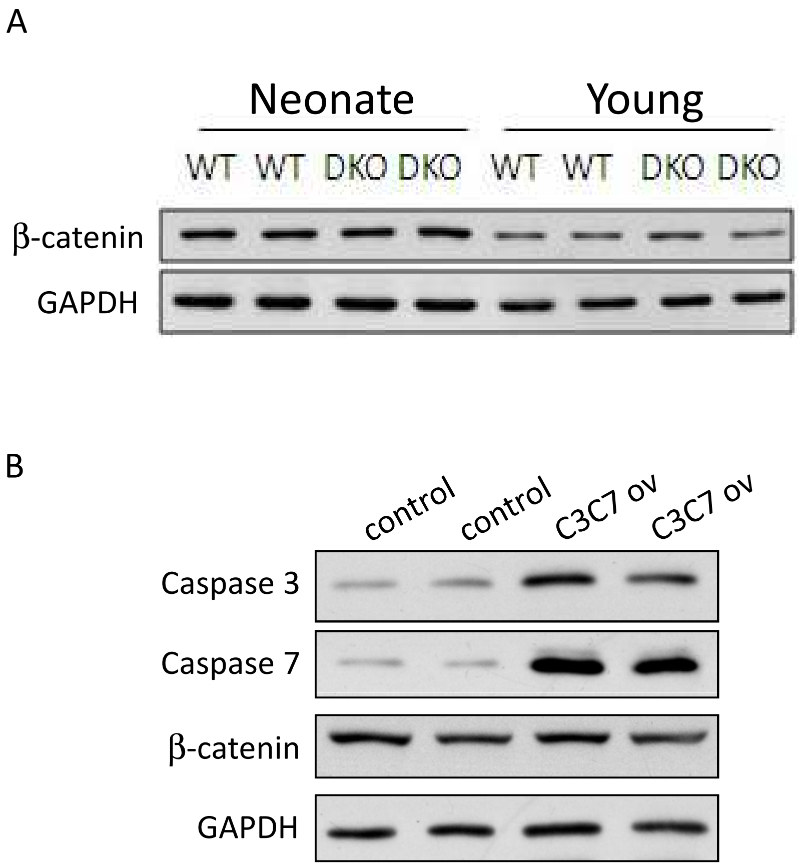

Beta-catenin expression is not regulated by executioner caspases in cardiomyocytes either *in vivo* or *in vitro*. A) Protein extracts from P1 neonates and P30 young wild type and caspase-3 and 7 double knockout mice were used to assess the expression of β-catenin (primary antibody: BD cat. № 610153 at 1/1000 dilution). WT: wild type; DKO: cardiomyocyte-specific caspase-3/caspase-7 double knockout mice. GAPDH was used to check even loading of samples at each age (n=2 per age and genotype). B) P2-4 postnatal rat cardiomyocytes were transduced with empty lentiviruses (control) or lentiviruses carrying vectors for caspase-3 and caspase-7 overexpression (C3C7 ov.) as reported in Materials and Methods section. Seventy-two hours later, β-catenin expression was assessed in total protein extracts from the cultures. A Western blot including two independent experiments per treatment is shown.

Figure G

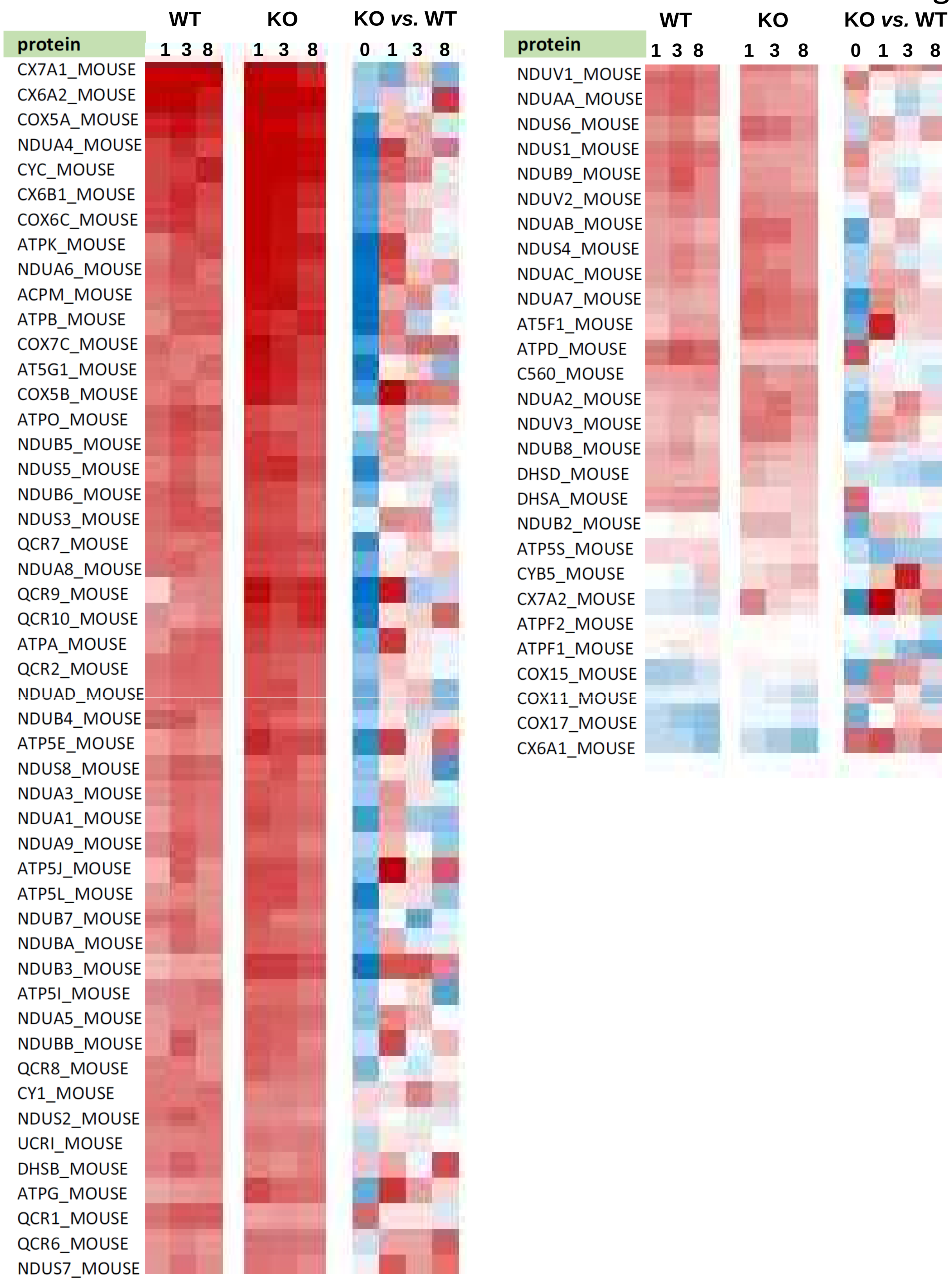

Identification of the proteins presented in Fig.3B. Relative abundance profiles of selected proteins of **Oxidative Phosphorylation** along development (0, 1, 3 and 8-month-old) in both wild type (WT) and caspase-3 and -7 knockout (KO) animals; in the left and middle columns, the changes are expressed separately for KO and WT animals in relation to the abundances at t=0, while in the rightmost column the abundance of proteins in the KO are compared with that of the WT animals at each time point.
